# *De novo* transcriptome assembly and identification of G-Protein-Coupled-Receptors (GPCRs) in two species of monogenean parasites of fish[Fn FN1]

**DOI:** 10.1051/parasite/2022052

**Published:** 2022-11-09

**Authors:** Víctor Caña-Bozada, F. Neptalí Morales-Serna, Emma J. Fajer-Ávila, Raúl Llera-Herrera

**Affiliations:** 1 Centro de Investigación en Alimentación y Desarrollo, A.C. Unidad Mazatlán en Acuicultura y Manejo Ambiental Mazatlán Sinaloa 82112 Mexico; 2 Instituto de Ciencias del Mar y Limnología, Unidad Académica Mazatlán, Universidad Nacional Autónoma de México Mazatlán Sinaloa 82040 Mexico

**Keywords:** Platyhelminthes, Dactylogyridae, Diplectanidae, Genomics, Proteins, Rhodopsin

## Abstract

Genomic resources for Platyhelminthes of the class Monogenea are scarce, despite the diversity of these parasites, some species of which are highly pathogenic to their fish hosts. This work aimed to generate *de novo*-assembled transcriptomes of two monogenean species, *Scutogyrus longicornis* (Dactylogyridae) and *Rhabdosynochus viridisi* (Diplectanidae), providing a protocol for cDNA library preparation with low input samples used in single cell transcriptomics. This allowed us to work with sub-microgram amounts of total RNA with success. These transcriptomes consist of 25,696 and 47,187 putative proteins, respectively, which were further annotated according to the Swiss-Prot, Pfam, GO, KEGG, and COG databases. The completeness values of these transcriptomes evaluated with BUSCO against Metazoa databases were 54.1% and 73%, respectively, which is in the range of other monogenean species. Among the annotations, a large number of terms related to G-protein-coupled receptors (GPCRs) were found. We identified 109 GPCR-like sequences in *R. viridisi*, and 102 in *S. longicornis*, including family members specific for Platyhelminthes. Rhodopsin was the largest family according to GRAFS classification. Two putative melatonin receptors found in *S. longicornis* represent the first record of this group of proteins in parasitic Platyhelminthes. Forty GPCRs of *R. viridisi* and 32 of *S. longicornis* that were absent in Vertebrata might be potential drug targets. The present study provides the first publicly available transcriptomes for monogeneans of the subclass Monopisthocotylea, which can serve as useful genomic datasets for functional genomic research of this important group of parasites.

## Abstract (fr)

Les ressources génomiques pour les Plathelminthes de la classe Monogenea sont rares, malgré la diversité de ces parasites dont certaines espèces sont hautement pathogènes pour leurs hôtes poissons. Ce travail visait à générer des transcriptomes assemblés *de novo* pour deux espèces de monogènes, S*cutogyrus longicornis* (Dactylogyridae) et *Rhabdosynochus viridisi* (Diplectanidae), fournissant un protocole pour la préparation de la bibliothèque d’ADNc avec des échantillons à faible apport utilisés en transcriptomique unicellulaire, ce qui a permis de travailler avec des quantités inférieures au microgramme d’ARN total avec succès. Ces transcriptomes se composent de 25 696 et 47 187 protéines putatives, respectivement, qui ont ensuite été annotées selon les bases de données Swiss-Prot, Pfam, GO, KEGG et COG. L’exhaustivité de ces transcriptomes évaluée avec BUSCO par rapport aux bases de données des Métazoaires était respectivement de 54,1 % et 73 %, ce qui est dans la gamme des autres espèces de monogènes. Parmi les annotations, un grand nombre de termes liés aux récepteurs couplés aux protéines G (RCPG) ont été trouvés. Nous avons identifié 109 séquences de type RCPG chez *R. viridisi* et 102 chez *S. longicornis*, y compris des membres de la famille spécifiques de Platyhelminthes. La rhodopsine était la plus grande famille selon la classification GRAFS. Deux récepteurs putatifs de la mélatonine trouvés chez *S. longicornis* représentent le premier signalement de ce groupe de protéines chez les Plathelminthes parasites. Quarante RCPG de *R. viridisi* et 32 de *S. longicornis*, qui sont absents chez les Vertébrés, pourraient être des cibles médicamenteuses potentielles. La présente sont fournit les premiers transcriptomes accessibles au public pour les monogènes de la sous-classe Monopisthocotylea, qui peuvent servir d’ensembles de données génomiques utiles pour la recherche génomique fonctionnelle de cet important groupe de parasites.

## Introduction

Monogenea is one of three classes of parasitic Platyhelminthes. The other two are Trematoda and Cestoda. All these are united under the monophyletic Neodermata, whereas the free-living platyhelminths, commonly termed planarians or turbellarians, are distributed in several other subtaxa [1]. There are two subclasses of monogeneans, Monopisthocotylea and Polyopisthocotylea, whose members are commonly ectoparasites of freshwater and marine fishes, but there are also members infecting internal organs of aquatic or semi-aquatic tetrapods. Between 3000 and 4000 species of monogeneans have been described [23], some of which have acquired economic relevance owing to their negative impact on finfish aquaculture [88]. Therefore, there is growing interest in increasing our understanding of the molecular mechanisms involved in host-parasite interactions.

A modern understanding of biology has come to rely on comparative approaches using genomic resources. In the case of trematodes and cestodes, genomes and transcriptomes have provided new and valuable insights into anthelmintic resistance and host–parasite interactions [79]. Unfortunately, the same level of progress has not been made with monogeneans. To date, the genomes of only three monogenean species (*Gyrodactylus bullatarudis*, *G. salaris*, and *Protopolystoma xenopodis*) have been published [21, 41, 61]. Transcriptomic data are scarcer still: the recently reported transcriptome of *Eudiplozoon nipponicum*, belonging to Polyopisthocotylea [104], is the sole example. This could be partly due to technical challenges, such as the small size of the organism (in the range of hundreds of micrometers) and the difficulty to obtain sufficient numbers of live individuals to purify the required amount of DNA or RNA. For instance, to assemble the genomes of *Gyrodactylus*, which has body lengths of 0.5–1 mm, Hahn et al. [41] used approximately 15,000 individuals and Konczal et al. [61] used 2000–3000 individuals. For the transcriptome of *E. nipponicum*, which has a much larger body than *Gyrodactylus*, Vorel et al. [104] required only 10 individuals.

The genes encoding G-protein-coupled receptors (GPCRs) are the largest family of genes in animal genomes. In fact, genomic studies on *G. bullatarudis* revealed that among duplicated genes, the most abundant group of Gene Ontology (GO) terms relate to GPCR signaling pathways [61]. GPCRs are evolutionarily conserved seven-transmembrane (TM) proteins with immense structural and functional diversity. Upon activation by various extracellular signals, they mediate many biological processes, including reproduction, locomotion and behavior [59, 69]. Some GPCRs are specific to platyhelminths and, therefore, their synthetic ligands have the potential to be parasite-selective anthelmintics [81]. Research in this area is of considerable significance because GPCRs are attractive and well-established drug targets. Of note, there are 475 drugs (~34% of all drugs approved by the FDA) that act on 108 unique GPCR targets [42].

In order to contribute to the genomics of monogeneans, the present work provides the *de novo* transcriptome analyses of two monogenean species, *Scutogyrus longicornis* (Dactylogyridae family) and *Rhabdosynochus viridisi* (Diplectanidae family), the first publicly available transcriptomes from Monopisthocotylea. Furthermore, the present work provides the first description of GPCRs in monogeneans. Dactylogyridae is the most diverse group of monogeneans in freshwater fishes. The dactylogyrid *S. longicornis* has mainly been observed in the freshwater Nile tilapia *Oreochromis niloticus* and other fish hosts such as *O. mortimeri*, *O. aureus*, and *Sarotherodon galilaeus* from different regions of the world [112]. Although this species has not been reported as pathogenic, *S. longicornis* and other dactylogyrids (*Cichlidogyrus* spp.) are frequently found in farmed tilapia [2, 52]. Members of the Diplectanidae family are commonly found to infect marine fishes, and some species are considered serious pathogens of farmed fish [3, 25]. In particular, *R. viridisi* is considered a threat to the production of the Pacific white snook, *Centropomus viridis* [17, 84]. The present study provides a comparative genomics framework for Diplectanidae monogeneans that should facilitate further studies aimed at testing evolutionary hypotheses in this underrepresented phylum.

## Materials and methods

### Parasite material

Adult individuals of *S. longicornis* were collected from the gills of tilapia (*Oreochromis niloticus*) cultured on a fish farm in the state of Sinaloa, northwestern Mexico. Adult individuals of *R. viridisi* were collected from the gills of snooks (*C. viridis*) reared in the marine fish hatchery at the Centro de Investigación en Alimentación y Desarrollo, Mazatlán, Sinaloa. The methods used for parasite sampling and species identification have already been described in detail by Caña-Bozada et al. [17, 18].

### RNA amplification and sequencing

RNA was extracted from 10 and 40 individuals of *S. longicornis* and *R. viridisi*, respectively. The methods used for RNA extraction, amplification, and sequencing are described in detail by Caña-Bozada et al. [17, 18]. The BioSamples accession numbers for *R. viridisi* and *S. longicornis* are SAMN17210467 and SAMN14607874, respectively. Briefly, for each species, individuals were rinsed in pure water, pooled, homogenized in 500 μL of 1× RNAshield (Zymo Research, Irvine, CA, USA), and vortexed with 100 mg of 200 μm glass with beads. RNA was extracted from pooled homogenates using a Quick-DNA/RNA Miniprep Kit (Zymo Research, Irvine, CA, USA), according to the manufacturer’s instructions. The obtained RNA fractions were below the limit of quantification (<1 ng/μL) when measured in a Qubit fluorometer using the HS dsDNA kit (Life Technologies, Carlsbad, CA, USA). Therefore, to obtain double-stranded full-length cDNA, 3 μL of purified total RNA was used for reamplification using a SMART-Seq v4 Ultra Low Input RNA Kit for sequencing (Takara Bio USA, Inc., San Jose, CA, USA), according to the manufacturer’s protocol. In addition, the cDNA was enriched over 12 cycles of PCR and then ultrasonically sheared into 300–500 bp fragments using a Covaris S220 ultrasonicator (Covaris, Woburn, MA, USA). The genomic libraries with multiplex adapters were prepared using a TruSeq Nano DNA Sample Preparation Kit (Illumina, San Diego, CA, USA), and run on a NextSeq500 Illumina platform (2 × 75 paired-end chemistry).

### *De novo* assembly and removal of nontarget sequences

The raw reads were processed in Trimmomatic v. 0.35 [10] to remove both low-quality reads and adapters (AVGQUAL: 20, MINLEN: 40). The cDNA paired reads were assembled using Trinity v. 2.8.6 [37] with default parameters (–kmer_size: 25), including the normalization step. The average coverage of the assembly was obtained with Bowtie2 v. 2.3.4 [66] and SamTools v. 1.7 [70].

Each assembly was aligned using BLASTx [15] against a database of protein sequences from bacteria, virus and fungi (downloaded from UniProt/Swiss-Prot; [4]), and reference proteins from the monogeneans *Gyrodactylus salaris* and *E. nipponicum,* the cestode *Hymenolepis microstoma,* the trematode *Schistosoma mansoni,* and the turbellarian *Schmidtea mediterranea* [46, 104] (https://github.com/jirivorel/Eudiplozoon-nipponicum-transcriptome-secretome). The contigs that were the best hits (*e*-values < 1e^−5^) with bacteria, virus or fungi were considered contaminant sequences and were discarded from the assemblies. Finally, to eliminate host contaminant sequences, each assembly was aligned using BLASTx against a database that included predicted proteins of the fish hosts *C. viridis* (NCBI SRA: SRP165941) and *O. niloticus* (NCBI Assembly: GCA_001858045.2), as well as the proteins of the aforementioned Platyhelminthes. The contigs that were best hits (*e*-values < 1e^−20^) with fish sequences were discarded from the assemblies. The filtered transcriptomes were used to predict ORFs and putative proteins.

### ORF prediction

The predicted ORFs were extracted from the assembled transcriptomes using TransDecoder 5.5.0 (http://transdecoder.github.io) [40], in combination with Swiss-Prot and Pfam searches (options: –retain_pfam_hits, –retain_blastp_hits) to predict and retain putative proteins longer than 100 amino acids. TransDecoder-predicted putative proteins with 100% identity were clustered using the CD-HIT 4.6 software [34] to reduce redundant sequences.

The putative proteins were filtered to eliminate possible contamination of bacteria, virus, fungi, and fish sequences. To this end, the predicted ORFs were compared against the EggNOG database [49] using the TRAPID tool [102]. EggNOG is a database containing information about orthologous relationships of prokaryotic, eukaryotic, and viral genomes. Hits with *e*-values < 1e^−5^ were considered significant. Some ORFs showed significant similarity with bacteria and fish sequences. In the case of *R. viridisi*, the bacterial ORFs belonged to *Vibrio* spp. Therefore, decontamination was performed: using BLASTp (*e*-values < 1e^−5^), the putative proteins of *R. viridisi* were aligned against proteins of *Vibrio sinaloensis*, *V. harveyi*, *V. brasiliensis, V. tubiashii* (NCBI Assembly IDs GCA_000189275.2, GCA_001558435.2, GCA_000189255.1, GCA_000772105.1, respectively), *C. viridis*, and the platyhelminths mentioned in the previous section*.* The putative proteins of *S. longicornis* were aligned using BLASTp (*e*-values < 1e^−5^) against protein sequences of *O. niloticus* and platyhelminths, and proteins of bacteria downloaded from the UniProt/Swiss-Prot database*.* Protein sequences for which the best hits were contaminant sequences were discarded.

In addition, stricter filtering was performed by aligning the translated proteins against the UniRef90 database using Diamond [12] with default parameters. The sequences that were best hits with Protostomia (taxid: 33317) were retrieved, and the remaining sequences were considered contaminants.

### Functional annotation

SignalP 4.1 [92] and TMHMM 2.0 [63] were used to predict signal peptides and TM domains in the putative proteins. Annotation of signal peptides, TM domains, and Swiss-Prot and Pfam data were loaded into Trinotate 3.1.1 (http://trinotate.github.io) [11]. Kyoto Encyclopedia of Genes and Genomes (KEGG) [54], Clusters of Orthologous Groups (COG) [101], and GO [35] IDs were retrieved. To avoid redundancy, in the Results section we show only the annotation of the longest protein for each gene, which was extracted using the script “get_longest_isoform_seq_per_trinity_gene.pl” in Trinity. Thus, each protein was considered a collection of expressed sequences of a putative gene [83]. In addition, the number of phenotypes (across worm model organisms) and UniProt keywords were determined with dcGO Predictor [31] using the Swiss-Prot IDs.

### Transcriptome quality assessment

Analysis of the completeness of assembly was performed using Benchmarking Universal Single-Copy Orthologs (BUSCO) v. 3.0.2 [106], using the core metazoan dataset, which contains 978 genes. The completeness of assembly was also verified with the TRAPID tool (conservation threshold = 0.9) using the core gene families of metazoans from the EggNOG database. Finally, we checked again for contamination using the EggNOG database and the TRAPID tool, as described above. As no reference genomes of *R. viridisi* and *S. longicornis* were available, BUSCO analyses were also performed on publicly available genomes or transcriptomes from other monogenean species (*G. salaris*, *E. nipponicum*, and *P. xenopodis*), and from other widely studied species of platyhelminths (*S. mansoni*, *H. microstoma*, *Macrostomum lignano*, and *S. mediterranea*). The obtained completeness values were used as references to evaluate the completeness of assembly for *R. viridisi* and *S. longicornis*.

### G-protein-coupled receptors

#### GPCR identification

To identify GPCRs, proteins were scanned against conserved domains of the different GPCR families obtained from the Pfam database (PF00001, PF00002, PF00003, PF01534, PF10320, PF10324, and PF10328), using HMMER 3.1b1 [32] with *e*-values < 1e^−5^. Then, TMHMM was used to detect TM domains; proteins with 3–15 TM domains were retained. Subsequently, the TM proteins were aligned using BLASTp (*e*-values < 1e^−4^) against annotated proteins from databases specific to platyhelminth species. To avoid overrepresentation of genes by their number of isoforms, the longest isoform for each gene was extracted using the Trinity helper script “get_longest_isoform_seq_per_trinity_gene.pl”. For comparative purposes, these analyses were also performed for the monogeneans *G. bullatarudis*, *G. salaris*, *E. nipponicum*, and *P. xenopodis*; the cestodes *T. asiatica* and *E. multilocularis*; the trematodes *Schistosoma japonicum*, *S. mansoni*, and *F. hepatica*; and the turbellarians *S. mediterranea* and *Bothrioplana semperi*. The reference proteins were obtained from the WormBase ParaSite database, except for *E. nipponicum* and *B. semperi*. The proteins of *E. nipponicum* were downloaded from https://github.com/jirivorel/Eudiplozoon-nipponicum-transcriptome-secretome [104]. The proteins of *B. semperi* were obtained using TransDecoder, with the options “–retain_pfam_hits” and “–retain_blastp_hits” from the assembly provided by Laumer et al. [67]. In addition, to remove possible remaining contaminant sequences and non-GPCR sequences, the GPCRs of *R. viridisi* and *S. longicornis* were aligned against the NCBI nonredundant protein database, including and excluding the phylum Platyhelminthes. For an initial annotation, the sequences of each predicted GPCR of *R. viridisi* and *S. longicornis* were aligned against reference sequences of GPCRs of *Caenorhabditis elegans*, *Bombyx mori*, *Homo sapiens*, *Lottia gigantea*, *Drosophila melanogaster*, *Daphnia pulex*, *Dictyostelium discoideum*, *Platynereis dumerilii*, *Anopheles gambiae*, *Lymnaea stagnalis*, *S. mediterranea*, and *S. mansoni*, using BLASTp. We classified GPCRs according to the GRAFS (glutamate, rhodopsin, adhesion, frizzled, secretin) system of classification [33], which groups receptors with shared evolutionary ancestry present in the Bilateria [111]. The rhodopsin family was further classified into the α subfamily, which contains amine, opsin-like, and melatonin receptors; and the β subfamily, which contains peptide and peptide hormone receptors.

To identify lineage-specific GPCRs, the predicted GPCRs of *R. viridisi* and *S. longicornis* were aligned against the NCBI nonredundant protein databases and limited by the option “Organism”, which included the sister classes Cestoda (taxid: 6199) and Trematoda (taxid: 6178), the basal class Rhabditophora (taxid: 147100), and the taxa Lophotrochozoa (taxid: 1206795; exclude: Platyhelminthes), Spiralia (taxid: 2697495; exclude: Lophotrochozoa), Protostomia (taxid: 33317; exclude: Spiralia), Bilateria (taxid: 33213; exclude: Protostomia), and Vertebrata (taxid: 7742). Predicted GPCRs with *e*-values > 1e^−5^ were considered to be specific for Monogenea, and were represented by heatmaps using the *ggtree* library in R (version 4.0.4). According to Kerfeld and Scott [60]

“*sequences with a recent shared ancestry will have a high degree of similarity; their alignments will have many identical residues, few substitutions and gaps, and tiny e-values. Conversely, sequences with an ancient common ancestor will be deeply divergent, with few shared sequence identities, many gaps, and larger e-values.*”

Thus, considering that hit sequences can be interpreted as sequences sharing evolutionary history, the log_10_-transformed *e*-values were correlated between different taxa using a Spearman analysis (Supplementary File S1). Correlation values close to 1 might indicate that monogenean GPCRs have similar evolutionary changes between the two respective taxa.

#### Phylogenetic analyses

To refine the GPCR annotation process for *R. viridisi* and *S. longicornis*, phylogenetic trees were constructed for each GPCR family with sequences used in the initial annotation*.* To this end, the proteins were aligned using MAFFT 7.31 [55] with the parameter “-genafpair” (E-INS-i), which is particularly useful for aligning proteins with conserved regions. The proteins belonging to the rhodopsin family, given their high number, were additionally trimmed with Trimal [19], using the parameter “-gappyout”, and their alignments refined in MUSCLE 3.8.31 [29]. The phylogenetic analysis was conducted with IQ-TREE [86], which performs a first step for the selection of the evolutionary model using the ModelFinder program [53] and subsequently the construction of the phylogenetic tree. The trees were constructed using 1000 replicates of the approximate likelihood ratio test, which is similar to the Shimodaira–Hasegawa test. Trees were visualized and annotated with FigTree 1.4.2 (available from http://tree.bio.ed.ac.uk/software/figtree/) and the *ggtree* library [110] using R. Proteins yielding contradictory results in different analyses were designated unclassified. Rhodopsin-family proteins that formed non-concordant groups were designated orphans. Information about the sequences used for the phylogenetic analysis of each GPCR family is presented in the Mendeley Data repository (DOI: https://doi.org/10.17632/2wvnwn4d7p.1), under the folder “phylogeny_secuences”.

## Results

Raw sequencing reads of *R. viridisi* and *S. longicornis* were deposited in the NCBI Sequence Read Archive (SRA) database under accession numbers SRR16891876 and SRR16889716, respectively (BioProjects PRJNA689569 and PRJNA625740). The *de novo* transcriptome assemblies and conceptual translations of *R. viridisi* and *S. longicornis* are available in the Mendeley Data repository (DOI:10.17632/2wvnwn4d7p.1) under the folder “assemblies_CDS_and_ORFs”.

### RNA samples and sequencing

A total of 271,701,941 and 80,972,372 raw paired-end reads of 75 bp were generated from *R. viridisi* and *S. longicornis* RNA samples, respectively. After removing low-quality reads (*Q* scores < 30) and adapters, 263,800,117 and 76,472,690 high-quality reads were obtained for the assemblies of *R. viridisi* and *S. longicornis*, respectively.

### *De novo* transcriptome assembly

For *R. viridisi*, a total of 392,252 contigs were assembled with an average coverage of 37.33× (SD = 172.693 bp), N50 of 3567 bp, and GC content of 45.72% (Table 1). The average contig length was 1235.85 bp. After the various decontamination procedures had been applied, 335,689 contigs with average length of 982 bp were retained in the assembly (N50 of 2132 bp, GC content 44.92%). This assembly contained 329,849,780 bp and 277,651 genes.

**Table 1:** Assembly and ORF statistics generated for *Rhabdosynochus viridisi* and *Scutogyrus longicornis*.

	***S. longicornis***							***R. viridisi***						
	Unfiltered	First filtering		Second filtering		Third filtering		Unfiltered	First filtering		Second filtering		Third filtering	
	Assembly	Assembly	ORF	Assembly	ORF	Assembly	ORF	Assembly	Assembly	ORF	Assembly	ORF	Assembly	ORF
# sequences ≥0 bp	51,817	49,873	22,197	48,775	21,241	47,814	19,291	294,928	269,985	55,141	267,005	47,497	264,494	41,059
# sequences ≥1000 bp	20,258	19,127	6663	14,119	6464	13,169	5997	63,638	54,716	8818	52,818	7067	51,163	5670
# sequences ≥3000 bp	10,291	9594	455	5361	445	4852	408	33,686	28,665	678	23,965	560	20,032	458
# sequences	82,366	78,594	29,315	68,255	28,150	65,835	25,696	392,252	353,366	64,637	343,788	55,124	335,689	47,187
# genes	48,086	46,372	13,179	45,995	12,821	45,194	12,020	313,109	284,085	30,291	280,648	26,854	277,651	23,857
Total length (bp)	111,966,103	105,481,027	23,584,962	70,992,660	22,763,481	66,069,113	20,893,392	484,764,415	422,751,986	40,917,888	369,417,136	34,017,756	329,849,780	28,346,136
GC (%)	42.56	42.49	46.38	42.49	46.44	42.46	4649	45.72	45.75	51.2	45.34	51.27	44.92	51.35
Average length of sequences	1359.37	1342.1	804.54	1040.11	808	1003.56	813	1235.85	1196.36	633.04	1074	617	982	600
N50	2906	2874	1020	2197	1029	2114	1038	3567	3496	750	2636	711	2132	666

For *S. longicornis*, a total of 82,366 contigs were assembled with an average coverage of 43.43× (SD = 197.105 bp), N50 of 2906 bp, and GC content of 42.56% (Table 1). The average contig length was 1359.37 bp. After decontamination, 65,835 contigs with an average length of 1003.56 bp were retained in the assembly (N50 of 2114 bp, GC content 42.46%). This assembly contained 66,069,113 bp and 45,194 genes.

### ORF prediction

We generated 64,637 ORFs for *R. viridisi* and 29,315 for *S. longicornis* (Table 1). The alignment of putative proteins of *R. viridisi* against *Vibrio* spp. and *C. viridis* sequences filtered out 9513 proteins (Table 2). The alignment of putative proteins of *S. longicornis* against bacteria and tilapia sequences filtered out 1165 proteins (Table 2). Finally, after alignment against sequences from Protostomia, 47,187 *R. viridisi* proteins encoded by 23,857 genes, and 25,696 *S. longicornis* proteins encoded by 12,020 genes, were retained*.* Information on the hits obtained from TRAPID is shown in Table 3.

**Table 2:** Information on contaminating sequences in the transcriptomes of *Rhabdosynochus viridisi* and *Scutogyrus longicornis*.

Filter		Contaminant taxa	Number of contaminant sequences	% GC	Average sequence	Number of bases
First filtering (assembly)	*S. longicornis* contigs (first filtering)	Bacteria	292	47.77	1362.50	397,850
		Tilapia	3285	43.16	1776.21	5,834,843
		Viruses and fungi	195	48.27	1319.91	258,703
	*R. viridisi* contigs (first filtering)	Bacteria	11,284	45.25	2085.79	23,536,059
		Snooks	27,133	45.50	1364.26	37,016,466
		Viruses and fungi	469	49.28	3112.80	1,459,904
Second filtering (ORF)	*S. longicornis* ORF (second filtering)	Bacteria	55	45.80	682.80	37,554
		Tilapia	1110	44.66	706.24	783,927
	*R. viridisi* ORF	*Vibrio* spp.	2071	45.99	621.04	1,286,169
		Snooks and tilapia	7442	51.93	754.36	5,613,963
Third filtering (ORF)	*S. longicornis* ORF (third filtering)	Non-Protostomia	2454	45.81	762.06	1,870,089
	*R. viridisi* ORF (third filtering)	Non-Protostomia	7937	50.9	714.58	5,671,620

**Table 3:** Numbers of top-hits of *Rhabdosynochus viridisi* and *Scutogyrus longicornis* ORFs matching to sequences of other species. Information was obtained from a similarity search analysis using TRAPID and the EggNOG database. Top hits obtained with sequences from other Platyhelminthes are shown.

*R. viridisi* unfiltered ORF		*R. viridisi* filtered ORF (first filtering: bacteria + fish)		*R. viridisi* filtered ORF (second filtering: bacteria + fish)		*S. longicornis* unfiltered ORF		*S. longicornis* filtered ORF (first filtering: bacteria + fish)		*S. longicornis* filtered ORF (second filtering: bacteria + fish)		*G. salaris*		*E. nipponicum*		*P. xenopodis*		*S. mediterranea*	
Hits	23,274	Hits	16,448	Hits	11,108	Hits	15,417	Hits	14,031	hits	12,273	Hits	8699	Hits	16,499	Hits	11,353	Hits	21,189
*Sm*	11,297 (48.54%)	*Sm*	9547 (58.04%)	*Sm*	7321 (65.91%)	*Sm*	8880 (57.6%)	*Sm*	8247 (58.78%)	*Sm*	7854 (63.99%)	*Sm*	4865 (55.93%)	*Sm*	10,583 (64.14%)	*Sm*	8327 (73.35%)	*Sm*	5756 (27.17%)
*On*	699 (3%)	*Bf*	455 (2.77%)	*Bf*	310 (2.79%)	*Bf*	466 (3.02%)	*Bf*	414 (2.95%)	*Bf*	330 (2.69%)	*Bf*	224 (2.58%)	*Bf*	417 (2.53%)	*Bf*	253 (2.23%)	*Bf*	1505 (7.1%)
*Bf*	608 (2.61%)	*Sp*	259 (1.57%)	*Sp*	174 (1.57%)	*Sp*	402 (2.61%)	*Sp*	377 (2.69%)	*Sp*	266 (2.17%)	*Sp*	123 (1.41%)	*Sp*	275 (1.67%)	*Sp*	199 (1.75%)	*Sp*	819 (3.87%)
*Ga*	431 (1.85%)	*On*	249 (1.51%)	*Dp*	135 (1.22%)	*Ap*	269 (1.74%)	*Ap*	214 (1.53%)	*Ap*	186 (1.52%)	*Dr*	119 (1.37%)	*Dp*	203 (1.23%)	*Nv*	107 (0.94%)	*Hm*	625 (2.95%)
*Sp*	396 (1.7%)	*Ga*	183 (1.11%)	*Is*	134 (1.21%)	*Tc*	214 (1.39%)	*Tc*	209 (1.49%)	*Tc*	185 (1.51%)	*Dp*	117 (1.34%)	*Nv*	192 (1.16%)	*Is*	104 (0.92%)	*Tc*	616 (2.91%)
*Vs*	353 (1.52%)	*Dr*	183 (1.11%)	*Tc*	117 (1.05%)	*Hm*	212 (1.38%)	*Hm*	201 (1.43%)	*Dp*	157 (1.28%)	*Tc*	116 (1.33%)	*Dr*	172 (1.04%)	*Dp*	99 (0.87%)	*Dp*	528 (2.49%)

Abbreviations: *Ap, Acyrthosiphon pisum; Bf, Branchiostoma floridae; Dp, Daphnia pulex; Dr, Danio rerio; Ga, Gasterosteus aculeatus; Hm, Hydra magnipapillata; Is, Ixodes scapularis; Nv, Nematostella vectensis; On, Oreochromis niloticus; Sm, Schistosoma mansoni; Sp, Strongylocentrotus purpuratus; Tc, Tribolium castaneum; Vs, Vibrio sinaloensis*.

**Figure 1:**
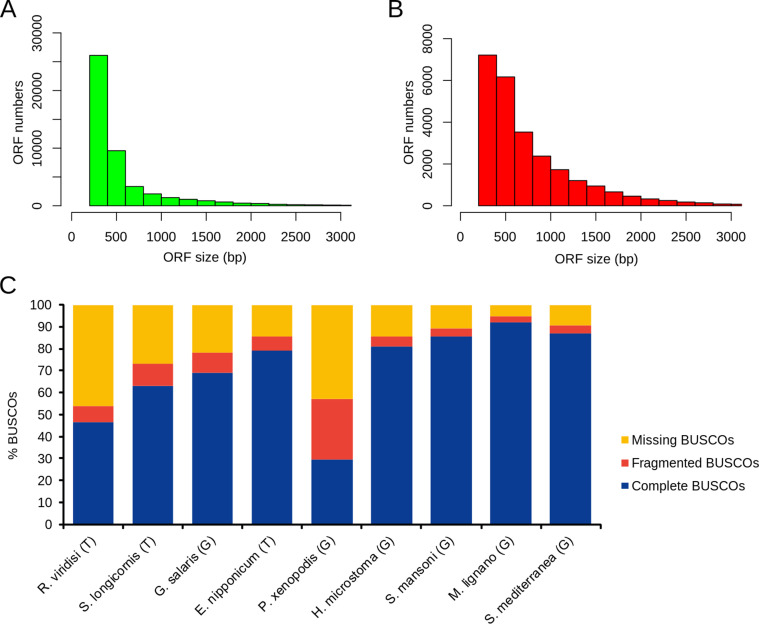
Sequence length distributions and assessment of completeness of *R. viridisi* and *S. longicornis* ORFs. (A–B) Sequence length distributions. (C) ORF completeness for *R. viridisi*, *S. longicornis* and other platyhelminths as determined by Benchmarking Universal Single-Copy Orthologous (BUSCO).

The average length of ORFs was 600 bp in *R. viridisi* and 813 bp in *S. longicornis*, in which 6128 (12.9%) and 6405 ORFs (24.9%) were longer than 1000 bp, respectively (Table 1). The distributions of ORF length are shown in Figures 1A–1B.

### Transcriptome quality assessment

BUSCO analysis of the putative proteins revealed 54.1% (46.6% completed, 7.5% fragmented) and 73% (63% completed, 10% fragmented) core metazoan genes for the *de novo* transcriptomes of *R. viridisi* and *S. longicornis*, respectively. The BUSCO completeness for the reference monogeneans was 57.3% for *P. xenopodis*, 78.1% for *G. salaris*, and 85.8% for *E. nipponicum*, whereas completeness for the other Platyhelminthes ranged between 81% and 92% (Fig. 1C). The TRAPID tool applied to the EggNOG database uncovered 54.9% and 73.6% core gene families in *R. viridisi* and *S. longicornis*, respectively.

### Functional annotation

Although all the putative proteins were annotated, we present results for the longest, nonredundant proteins to avoid overrepresentation of sequences. Complete results are shown in Supplementary Tables S1–S3. After removing redundant sequences (isoforms), the representative proteins were reduced in number from 47,187 to 23,857 in *R. viridisi*, and from 25,696 to 12,020 in *S. longicornis.* For *R. viridisi*, 3214, 2761, 2798, 2114, and 1205 protein sequences were aligned to the Swiss-Prot, Pfam, GO, KEGG, and COG databases, respectively. In addition, 6824 proteins were predicted to contain a TM domain, and 2823 a signal peptide. For *S. longicornis*, 5849, 5422, 5647, 4132, and 2074 proteins were aligned to the Swiss-Prot, Pfam, GO, KEGG, and COG databases, respectively. In addition, 2579 proteins were predicted to contain a TM region, and 1098 a signal peptide. A general overview of the functional and ontological representation is presented in Supplementary File S1 and Supplementary Figs. S1–S4.

### G-protein-coupled receptors

GO terms relating to GPCRs were among the most abundant in both Biological Process and Molecular Function terms (Supplementary Figs. S1, S2). We identified 109 GPCR-like sequences in *R. viridisi* and 102 in *S. longicornis*, of which at least 40% contained seven-TM domains. An analysis of all sequences with at least three domains revealed that the putative GPCRs comprised one glutamate, 99 rhodopsin, two adhesion, six frizzled, and one secretin in *R. viridisi*; and three glutamate, 94 rhodopsin, one adhesion, three frizzled, and one secretin in *S. longicornis.* Fifteen and nine putative GPCRs presented signal peptides in *R. viridisi* and *S. longicornis*, respectively; mainly, these were proteins with seven-TM domains. Information about GPCR identification in *R. viridisi* and *S. longicornis* is presented in Supplementary Figs. S1, S2. In addition, for comparative purposes, we predicted the GPCRs of other species of monogeneans, trematodes, cestodes, and turbellarians. Ninety GPCRs were identified in *G. bullatarudis*, 98 in *G. salaris*, 85 in *P. xenopodis*, 23 in *E. nipponicum*, 105 in *F. hepatica,* 117 in *S. japonicum,* 118 in *S. mansoni*, 70 in *E. multilocularis*, 79 in *T. asiatica*, 67 in *B. semperi*, and 384 in *S. mediterranea* (Table 4)*.* Information about GPCR identification in these platyhelminths is provided in Supplementary Table S5.

**Table 4:** Identification and classification of GPCR obtained from different platyhelminths.

	Monogenea						Trematoda			Cestoda		Rhabditophora	
	Monopisthocotylea				Polyopisthocotylea		Digenea			Eucestoda		Seriata	
GPCR family	*R. viridisi*	*S. longicornis*	*G. bullatarudis*	*G. salaris*	*P. xenopodis*	*E. nipponicum*	*S. japonicum*	*S. mansoni*	*F. hepatica*	*T. asiatica*	*E. multilocularis*	*B. semperi*	*S. mediterranea*
Adhesion/secretin	2/1	1/1	5	4		7	6	7		4	4	13	27
Frizzled	6	3	9	9	9	2	7	6	5	5	6	7	11
Glutamate	1	3	3	2	3		4	3	3	6	2	2	10
Rhodopsin	99	94	82	83	73	14	100	102	97	64	58	45	336
Total	110	102	99	98	85	23	117	118	105	79	70	67	384

**Figure 2:**
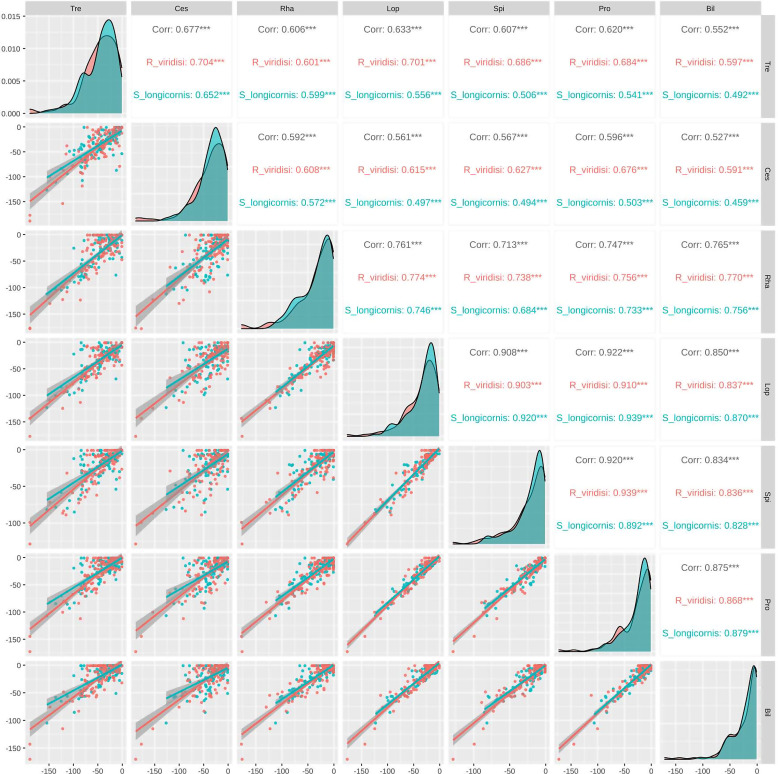
Spearman analysis. *E*-values were highly correlated between non-platyhelminth taxa (*r* > 0.834, *p* < 0.001), whereas *e*-values between platyhelminth taxa were less correlated (*r* < 0.677, *p* < 0.001).

The alignments of monogenean GPCRs against those of Trematoda, Cestoda, and Rhabditophora gave lower *e*-values than with other taxa. According to the Spearman analysis, the *e*-values were highly correlated between non-platyhelminth taxa (*r* > 0.834, *p* < 0.001) but were less correlated between platyhelminth taxa (*r* < 0.677, *p* < 0.001) (Fig. 2 and Supplementary Table S5).

**Figure 3:**
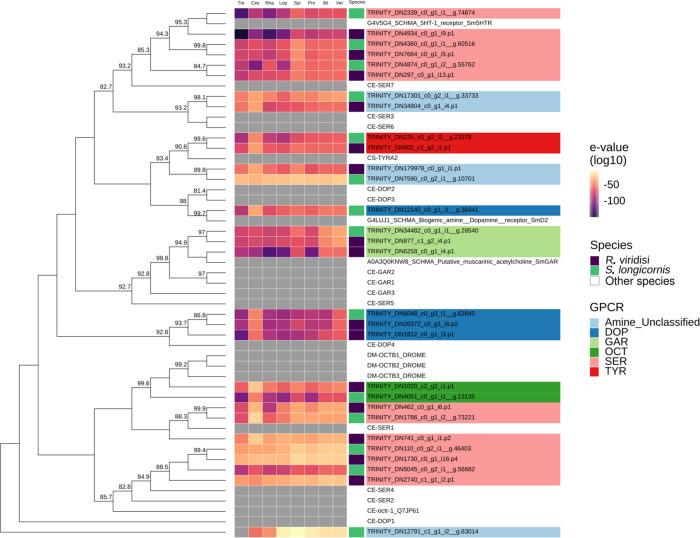
Phylogenetic analysis of *Rhabdosynochus viridisi* and *Scutogyrus longicornis* amine-subfamily GPCRs. The midpoint-rooted phylogenetic tree was constructed using 1000 replicates of the approximate likelihood ratio test (similar to the Shimodaira–Hasegawa test). The LG + F + G4 model was implemented. The colored boxes indicate the log_10_-transformed *e*-values obtained from the alignment of the *R. viridisi* and *S. longicornis* sequences against sequences of different taxa using the NCBI database (Tre, Trematoda; Ces, Cestoda; Rha, Rhabditophora; Lop, Lophotrochozoa; Spi, Spiralia; Pro, Protostomia; Bil, Bilateria; Ver, Vertebrata); the species to which the sequence belongs (*R. viridisi* or *S. longicornis*); and the type of receptor inferred from phylogenetic similarity to reference sequences. Only nodes with bootstrap support greater than 80 are shown.

**Figure 4:**
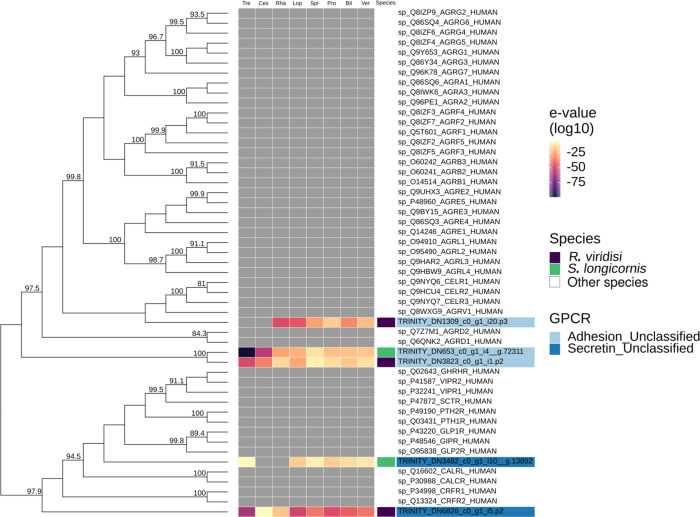
Phylogenetic analysis of *Rhabdosynochus viridisi* and *Scutogyrus longicornis* adhesion/secretin-subfamily GPCRs. The midpoint-rooted phylogenetic tree was constructed using 1000 replicates of the approximate likelihood ratio test (similar to the Shimodaira–Hasegawa test). The VT+R5 model was implemented. The colored boxes indicate the log_10_-transformed *e*-values obtained from the alignment of the *R. viridisi* and *S. longicornis* sequences against sequences of different taxa using the NCBI database (Tre, Trematoda; Ces, Cestoda; Rha, Rhabditophora; Lop, Lophotrochozoa; Spi, Spiralia; Pro, Protostomia; Bil, Bilateria; Ver, Vertebrata); the species to which the sequence belongs (*R. viridisi* or *S. longicornis*); and the type of receptor inferred from phylogenetic similarity to reference sequences. Only nodes with bootstrap support greater than 80 are shown.

**Figure 5:**
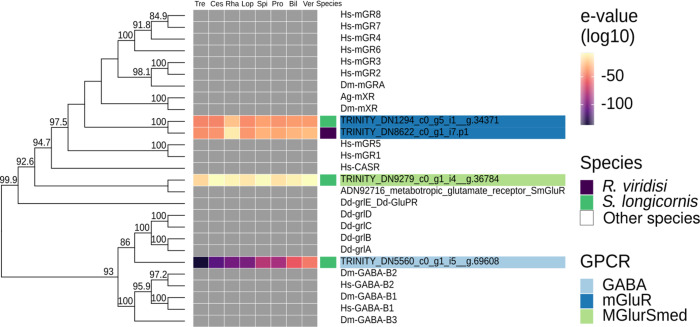
Phylogenetic analysis of *Rhabdosynochus viridisi* and *Scutogyrus longicornis* glutamate-subfamily GPCRs. The midpoint-rooted phylogenetic tree was constructed using 1000 replicates of the approximate likelihood ratio test (similar to the Shimodaira–Hasegawa test). The LG+F+R5 model was implemented. The colored boxes indicate the log_10_-transformed *e*-values obtained from the alignment of the *R. viridisi* and *S. longicornis* sequences against sequences of different taxa using the NCBI database (Tre, Trematoda; Ces, Cestoda; Rha, Rhabditophora; Lop, Lophotrochozoa; Spi, Spiralia; Pro, Protostomia; Bil, Bilateria; Ver, Vertebrata); the species to which the sequence belongs (*R. viridisi* or *S. longicornis*); and the type of receptor inferred from phylogenetic similarity to reference sequences. Only nodes with bootstrap support greater than 80 are shown.

**Figure 6:**
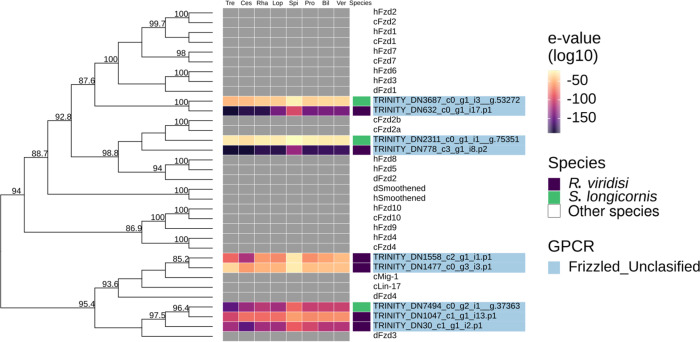
Phylogenetic analysis of *Rhabdosynochus viridisi* and *Scutogyrus longicornis* frizzled-subfamily GPCRs. The midpoint-rooted phylogenetic tree was constructed using 1000 replicates of the approximate likelihood ratio test (similar to the Shimodaira–Hasegawa test). The WAG + F + R5 model was implemented. The colored boxes indicate the log_10_-transformed *e*-values obtained from the alignment of the *R. viridisi* and *S. longicornis* sequences against sequences of different taxa using the NCBI database (Tre, Trematoda; Ces, Cestoda; Rha, Rhabditophora; Lop, Lophotrochozoa; Spi, Spiralia; Pro, Protostomia; Bil, Bilateria; Ver, Vertebrata); the species to which the sequence belongs (*R. viridisi* or *S. longicornis*); and the type of receptor inferred from phylogenetic similarity to reference sequences. Only nodes with bootstrap support greater than 80 are shown.

Nine and four putative proteins (peptide and orphan) of *R. viridisi* and *S. longicornis*, respectively were found to be specific to the Platyhelminthes (*e*-values ≥ 1e^−5^). Of these, six peptides and one orphan receptor were specific to parasitic taxa. Five peptide receptors of *R. viridisi* and one orphan receptor of *S. longicornis* were specific to Monogenea. We also found that of the *R. viridisi* and *S. longicornis* GPCRs, 23 were specific to Lophotrochozoa, 31 to Spiralia, and 64 to Protostomia. Thirty-seven (37) *R. viridisi* and 27 *S. longicornis* proteins (peptide and orphan) were absent in Bilateria; 40 and 32 from the respective species were absent in Vertebrata. Heatmaps showing monogenean-specific GPCRs are presented in Figures 3–6 and Supplementary Figs. S7–S8.

*Rhabdosynochus viridisi* and *S. longicornis* shared 100 and 99 GPCRs, respectively with Trematoda, 96 and 92 with Cestoda, 85 and 88 with free-living Platyhelminthes, and 98 and 97 with Lophotrochozoa. Furthermore, we found that four proteins (peptide and adhesion) of *R. viridisi* and *S. longicornis* were absent in parasitic platyhelminths but present in their free-living counterparts.

#### Rhodopsin family

Rhodopsins formed the largest family of the GRAFS classifications in all the species (Table 4). We identified 99 and 94 rhodopsin members in *R. viridisi* and *S. longicornis*, respectively. Thus, rhodopsin represented >90% of the putative GPCR proteins. The phylogenetic analysis revealed two subfamilies (*α* and *β*) and four subgroups (amine, opsin, peptide and melatonin receptors) of rhodopsins (Supplementary Figs. S5 and S6). In *R. viridisi*, the α subfamily comprised 15 amine receptors, three opsins and one melatonin; the β subfamily contained 72 peptide receptors. In *S. longicornis*, the α subfamily comprised 14 amine receptors, nine opsins and two melatonins; the *β* subfamily contained 62 peptide receptors. Eight receptors of *R. viridisi* and seven of *S. longicornis* could not be grouped into any subfamily and were considered orphan receptors. Nucleotide-activated receptors (*γ* subfamily) and olfactory receptors (*δ* subfamily) were absent in both species.

A separate phylogenetic analysis was performed for amine and peptide receptors and included reference proteins from other organisms (Fig. 3; Supplementary Figs. S7 and S8). The analysis of the amine receptor group showed that proteins of both *R. viridisi* and *S. longicornis* clustered with octopamine (OCT1/2/3 of *Drosophila melanogaster*), dopamine (DOP4 of *C. elegans*), G-protein-coupled acetylcholine receptor (GAR1/2 of *C. elegans*, SmGAR of *S. mansoni*), tyramine (TYR2 of *C. elegans*), and serotonin (SmSER5HT-1 of *S. mansoni*, SER1 and SER4 of *C. elegans*); and a protein of *S. longicornis* clustered with dopamine (SmD2 of *S. mansoni*) (Fig. 3). Analysis of the peptide receptor group identified clusters with allatostatin B (AstB of *L. gigantea* and *D. melanogaster*), FMRFamide (FMRFa of *D. melanogaster*), luqin/RYamide (Luq/RYamide of *P. dumerilii*, *L. stagnalis*, *L. gigantea*, and *D. melanogaster*), myosupressin/myomodulin (MS/MM of *P. dumerilii*, *B. mori*, and *D. melanogaster*), neuropeptide KY (NKY of *P. dumerilii*), neuropeptide Y (NPY of *P. dumerilii*, *H. sapiens*, *L. gigantea*, and *D. melanogaster*), and thyrotropin-releasing hormone/EFLG family (TRH-EFLG of *P. dumerilii*, *H. sapiens*, *L. gigantea*, and *D. pulex*) in *R. viridisi* and *S. longicornis*. Within NPY, a group of six proteins clustered with NPYR-1 of *S. mediterranea*. Allatotropin/orexin (AT/orexin of *B. mori*, *H. sapiens*, and *L. gigantea*), neuromedin U family (NMU of *H. sapiens*, *L. gigantea*, and *D. melanogaster*), SIFamide/neuropeptide FF (SIFa-NPFF of *H. sapiens*, *L. gigantea*, and *D. melanogaster*), and tachykinin (TK of *H. sapiens*, *L. gigantea*, and *D. melanogaster*) receptors were found only in *R. viridisi.*

#### Adhesion and secretin families

Phylogenetic analysis of the adhesion and secretin families showed that *R. viridisi* and *S. longicornis* have non-orthologous receptors in humans. Nonetheless, the GPCRs were classified into adhesion and secretin receptors, with two adhesion receptors in *R. viridisi* and one in *S. longicornis,* and one secretin in each species (Fig. 4).

#### Glutamate family

The glutamate family included one protein of *R. viridisi* and three of *S. longicornis*, which were clustered with metabotropic glutamate receptors (mGluRs) and mGluR-like proteins of *H. sapiens*, *D. melanogaster*, and *A. gambiae.* One protein of *S. longicornis* grouped with GABA B of *H. sapiens*, *D. melanogaster*, and *D. discoideum*, and another clustered with a mGluR of *S. mediterranea* (SmGluR) (Fig. 5).

#### Frizzled family

We identified nine frizzled family receptors (six in *R. viridisi*, three in *S. longicornis*), seven of which presented signal peptides. The phylogenetic analysis clustered these proteins with fzd2a/b of *C. elegans* and *D. melanogaster*, and fzf5/8 of *H. sapiens* (Fig. 6)*.* Other clusters were formed by one frizzled protein from *R. viridisi* and *S. longicornis* and fzd1/2/3/6/7 of *C. elegans*, *H. sapiens*, and *D. melanogaster*. The remainder of the proteins showed discordance between phylogenetic analysis and alignment with BLAST, and they were therefore not classified.

## Discussion

The present study provides the first publicly available transcriptomes for monogeneans of the subclass Monopisthocotylea. These parasites are typically small: adult worms of *R. viridisi* and *S. longicornis* have a body length of approximately 400 μm. Thus, one of the main challenges of the present study was to obtain enough genomic material for sequencing. This difficulty might explain why genomic data for monogeneans are scarce. Another challenge was the filtering-out of contaminant sequences that belong mainly to bacteria and fish. Contaminant sequences were removed using multiple filtering steps that involved the use of tools such as TRAPID. This program validated our decontamination strategy, because most ORFs matched with sequences of *S. mansoni*. Similar results were observed when we used TRAPID on protein-coding sequences of *G. salaris*, *E. nipponicum*, *P. xenopodis*, *S. mansoni*, *H. microstoma*, *M. lignano*, and *S. mediterranea*. It is important to note that our filtering strategy included data from the monogeneans *G. salaris*, *P. xenopodis*, and *E. nipponicum*, which allowed us to reduce the incidence of false negatives. Most of the putative proteins of *R. viridisi* and *S. longicornis* have not been characterized previously, which is consistent with the transcriptome of *E. nipponicum* [104]. Among the few annotated proteins, we found abundant terms related to GPCRs, as well as terms related to other membrane proteins, proteases, and kinases.

The quality of our assemblies can be considered adequate, because for non-model organisms the reported completeness is typically between 50% and 95%, whereas for model organisms it is higher than 95% [98]. In addition, we verified that the completeness of the transcriptomes or publicly available genomes of other monogeneans was between 57.3% and 85.8%, which is a similar range to that for *R. viridisi* and *S. longicornis*.

### G-protein-coupled receptors

Phylogenetic analysis and inspection of each sequence allowed us to predict and classify the GPCRs of *R. viridisi* and *S. longicornis* with a high degree of confidence. Monogenean GPCRs presented higher sequence similarity with other parasitic Platyhelminthes, mainly trematodes, than with other taxa. However, most receptors are present in non-platyhelminth ancestral taxa. This is consistent with the findings of Koziol et al. [62], who argued that most GPCRs in one taxon are orthologs of GPCRs from ancestral taxa. According to McVeigh et al. [82], the majority of GPCRs are expressed and found in the RNA-Seq datasets. For instance, with the support of RNA-Seq data, Campos et al. [16] identified 65 of the 70 identified GPCRs in the genome of *Schistosoma haematobium*, and 56 of the 68 in the genome of *S. mansoni*. In the present study, RNA-Seq was performed on adult parasites. Therefore, the identification of GPCRs represents an approximation of the GPCRs present in the genomes of monogeneans. In addition, the numbers of putative GPCRs in *R. viridisi* and *S. longicornis* are comparable to those predicted for the other monopisthocotylean monogeneans and trematodes that we analyzed (Table 4). Konczal et al. [61] also found that GPCR signaling pathways were among the most represented terms in the monogenean *G. bullatarudis*. In general, GPCRs act in neuromuscular signaling, chemosensation, and development [9, 76]. However, to the best of our admittedly limited knowledge on GPCRs in flatworms, only 11 flatworm receptors have been deorphanized and the functionality of even fewer has been determined [81].

By aligning GPCRs of monogeneans with other platyhelminth and non-platyhelminth taxa, a higher correlation was expected between *e*-values of Platyhelminthes; however, the correlation was higher between non-Platyhelminthes. This might indicate that the GPCRs are more diverse within Platyhelminthes. That is consistent with the work of Koziol et al. [62], who reported that most GPCRs from parasitic flatworms are quite diverse in comparison with those in other bilaterians. Such divergence might pose a problem for classifying these GPCRs [111]. Therefore, in addition to robust classification studies, experimental validation through the identification of ligands is recommended. However, *in silico* analysis can help us further understand the evolution of these receptors.

The dominance of rhodopsin receptors found in the present study, including members specific to monogeneans or Platyhelminthes, is consistent with findings in species from different phyla [33, 82, 111, 113]. McVeigh et al. [82] noted that the rhodopsin family includes highly diverse flatworm-specific receptors. In parasitic platyhelminths, these receptors might have key roles, such as in determining virulence and in host finding [71, 111]. Rhodopsin receptors are stimulated by light, odorant molecules, and neurotransmitters, and perform functions in vision and olfaction [71]. The rhodopsin family was mostly represented by the β subfamily, predominantly peptide receptors. Some ligands of peptide receptors are neuropeptides – intercellular signaling molecules that act as neurotransmitters, neuromodulators, or neurohormones [30]. Neuropeptide signaling systems in flatworms are associated with locomotion, reproduction, feeding, and larval host finding [78, 80]. The two putative melatonin receptors found in *S. longicornis* represent the first record of this group of proteins in parasitic platyhelminths. These receptors were orthologous to the melatonin receptors of *S. mediterranea*. In free-living freshwater flatworms, melatonin receptors have been implicated in the control of biological rhythms, with neoblast proliferation processes, and posttraumatic regeneration [85, 109]. It is possible that melatonin receptors regulate egg-laying rhythms in monogeneans, which leads to increased egg production in periods of darkness [44, 75].

There were some members of the glutamate family in *R. viridisi* and *S. longicornis*. This family is associated with the modulation of glutamate responses for a variety of central nervous system functions [8]. In vertebrates, the glutamate GPCRs comprise subgroups with extremely divergent physiological roles owing to expansion during vertebrate evolution. However, some of these subgroups are missing in invertebrates [8]. The mGluR and GABA receptors are two of the few subgroups to be retained in invertebrates and in most bilateral species [8]. The Platyhelminthes have mGluR; however, GABA receptors have yet to be reported [82, 111]. Similar to other platyhelminths, we found mGluR in *R. viridisi* and *S. longicornis.* Although parasitic platyhelminths seem to have lost receptors from their free-living ancestor, we found one probable GABA receptor in *S. longicornis*. It is expected that further studies will confirm the presence of GABA receptors in monogeneans.

Furthermore, adhesion and secretin GPCRs were found in *R. viridisi* and *S. longicornis*. The adhesion family has protein domains in the extracellular region that participate in cell–cell interactions and are present in almost every organ system with physiological roles in development, immunity, reproduction, epithelial and neuronal function, and tumorigenesis [65]. Although in vertebrates this family has the second most members, in invertebrates, especially parasitic platyhelminths, it has few members [111]. The secretin GPCRs mediate important hormonal functions through binding those hormones, and it has been suggested that they originate from the adhesion GPCRs [87], owing to the similarity of sequence and the large number of proteins in this family. However, we found a similarly low number of secretin and adhesion GPCR sequences in Platyhelminthes, which indicates that adhesion GPCRs were lost as this taxonomic group evolved.

Frizzled receptors in *R. viridisi* and *S. longicornis* were not classified at the subfamily level. Pathways involving frizzled receptors have important roles in adult tissues and developing embryos in the regulation of cell polarity and formation of neural synapses; specifically, they interact with Wnt proteins and other ligands [47]. In comparison with Platyhelminthes of the classes Cestoda and Rhabditophora, in monogeneans no smoothened receptors (SMOs) were found [82]. SMO is a key signal transducer in the hedgehog (Hh) pathway, which is important in development [5]. Although SMO is present in both invertebrates and vertebrates, its domains are divergent between lineages, which might explain the differences in SMO signaling in different organisms [6].

### GPCR drug targets

The 40 GPCRs of *R. viridisi* and the 32 of *S. longicornis* that were absent in Vertebrata could be considered potential drug targets. Given their role as mediators of signal transduction involving a range of neurotransmitters, GPCRs have been proposed as drug targets in parasitic platyhelminths [81]. We assumed that some functions of the monogenean GPCRs are similar to those found in other helminths. The three monogenean GPCRs clustered with NPYR-1 of *S. mediterranea* might be involved in sexual maturation and germ cell differentiation [96]. The monogenean GPCR that was orthologous to the dopamine receptor SmD2 of *S. mansoni* might be a component of the neuromuscular system [100]. The monogenean GPCRs similar to SmSER5HT-1, expressed in nerve tissue, and SmGAR of *S. mansoni*, and also those similar to DOP-1 of *C. elegans*, might be involved in motility [20, 74, 91]. The monogenean GPCRs clustered with octamine in *D. melanogaster*, as well as those clustered with SER-1 of *C. elegans* might participate in the activation of multiple signaling pathways to induce egg laying [20, 68, 72]. The potential of GPCRs as drug targets has been shown in other parasites. For instance, Santos et al. [97] showed that knockout of the GPCR-like PfSR25 increases the susceptibility of malaria parasites to antiparasitic compounds. In the future, in the context of drug repurposing, it would be necessary to examine the role of GPCRs in the susceptibility of monogeneans to existing drugs.

### Other membrane proteins

In addition to GPCRs, we revealed the important presence of putative proteins related to membrane trafficking and transport. These proteins are necessary for exocytosis, endocytosis, endosome–lysosome transport, endosome–Golgi transport, endoplasmic reticulum–Golgi transport, autophagy, and so forth. In parasitic platyhelminths, these functions are important for host–parasite interactions as well as xenobiotic detoxification. For instance, the excretory/secretory proteins released by parasites facilitate feeding and modulation of host immune responses [28, 71]. TM proteins such as the ABC transporters play a principal role in the export of a wide spectrum of different substrates. It has been suggested that in helminths, several ABC transporters are involved in drug resistance and detoxification processes to facilitate survival in the host [64].

Innexins were among the most abundant proteins in *R. viridisi* and *S. longicornis*. Innexins are integral membrane proteins that participate in cellular communication in invertebrates [24]. These proteins are bifunctional, that is, they can form gap junctions and unpaired membrane channels (innexons). Gap junctions allow the diffusion of second messengers and other ions and small molecules between two adjacent cells, whereas innexons allow the exchange of ions and metabolic and signaling molecules between the cell interior and the extracellular milieu [24, 39]. The number of innexins found herein is similar with other species of platyhelminths: 24 in *Taenia solium*, 25 in *S. mansoni*, 24 in *S. japonicum*, 17 in *H. microstoma*, 19 in *E. multilocularis*, and 20 in *Echinococcus granulosus* [103]. This diversity of innexins reflects their involvement in several biological processes, such as morphogenesis, neurogenesis, behavior, memory, and the immune response [39]. Some gap junction proteins might be lineage-specific. For example, CX39.4 is a teleost lineage-specific gap junction protein involved in the formation of skin patterns [105]. Oviedo et al. [90] reported the involvement of specific innexins in tissue regeneration in planarians. Thus, the innexins found in monogeneans might become the focus for experiments aimed at discovering lineage-specific functions.

The ion channel pathways, principally those involving voltage-gated channels, were highly represented in both monogeneans studied. Ion channels are multimeric complexes of membrane proteins involved in the passive diffusion of ions across biological membranes. These proteins perform key functions in the nervous system [48]. Platyhelminths have relatively well-developed neuromuscular systems, which coordinate many activities essential for parasite survival, such as motility, feeding, excretion, and egg laying [94, 107]. The voltage-gated potassium (Kv) and calcium (Cav) channels are well represented in the genomes of flatworms [107]. In schistosomes, activated Cav channels initiate muscle contraction and are associated with synaptic transmission, enzyme activity, and gene expression. It is possible that these proteins perform similar functions in monogeneans.

### Proteases

Cysteine cathepsins, especially cathepsins L, were annotated in *S. longicornis* and *R. viridisi*. It is thought that cathepsins L perform functions associated with the modulation or impairment of host immune responses, given their ability to degrade IgG and host Toll-like receptors [14, 22]. However, proteases in general have been scarcely studied in monogeneans [93]. Recently, Jedličková et al. [51] reported evidence for the involvement of a variety of cathepsins L in several processes requiring proteolysis in *E. nipponicum*. Furthermore, these authors observed that some cathepsins L of this monogenean can cleave immunoglobulins *in vitro*, which might form part of the mechanism of host immune evasion. Other parasitic platyhelminths have also been found to possess a variety of cathepsins L. For instance, Robinson et al. [95] suggested that the expansion of the cathepsin L family in *F. hepatica* is related to its ability to infect and adapt to new hosts. A better understanding of the biology of monogeneans, including their interactions with hosts, will likely require rigorous molecular and biochemical characterization of their cysteine cathepsins.

### Kinases

We found high representation of pathways involving kinase activity, mainly that of serine/threonine kinases. Kinases mediate most of the signal transduction occurring in cells and thus control many cellular processes such as metabolism, transcription, cell cycle progression, cytoskeletal rearrangement and cell movement, apoptosis, and differentiation [77]. In helminths, kinases, such as the serine/threonine kinases, have functions associated with growth and development [27]. Nonetheless, this group of proteins have scarcely been studied in platyhelminths. Evidence that polo-like kinases participate in the regulation of the cell cycle in both mitosis and meiosis in *S. mansoni* [26] indicates a role at the schistosomula stage and association of these kinases with rapid growth and body remodeling [38]. Members of the protein kinase C (PKC) family possibly have a central function in secretion and larval transformation, and influence the host’s response to the parasite by directly regulating interactions with host cells [7]. Furthermore, the differential expression of kinases throughout the development of *S. mansoni* and *F. hepatica* suggests they perform important functions [38, 45]. Kinases are well-known drug targets in humans [89] and are now being investigated as targets for new anthelmintics [36].

### Phenotype

The proteins of *R. viridisi* and *S. longicornis* participate mainly in the following phenotypes: negative chemotaxis variant and chemotaxis, protein phosphorylation, egg-laying, and drug-induced gene expression variants. Being ectoparasites, monogeneans are directly affected by environmental changes, so these parasites must respond effectively to these changes to survive. In the context of medicine, the drug-induced gene expression phenotype might be interesting to study in monogeneans because changes in genes or in gene expression in response to anthelmintics can lead to drug resistance and survival of the parasite [50]. The development of drug resistance has already been observed in helminths, including monogeneans [13, 108]. According to WormBase, the chemotaxis variant is related to the direct movement of an animal in response to chemical repellents with pathways that involve GPCRs and kinase, and calcium channel activity. In monogeneans, chemotaxis is essential for host finding and egg hatching [56, 58]. In *C. elegans*, changes in chemical signals corresponding to food levels and population density in the environment are either directly received by GPCRs or interact with GPCR pathways, and can thus regulate the stage of animal development [73, 99]. The egg-laying variant is associated with variations in the stage at which eggs are laid, egg-laying cycle, number of eggs laid, or egg laying in response to stimuli. This phenotype involves cytoskeletal proteins. Monogeneans are highly prolific, and they are able to maintain egg production in a wide range of water temperatures [43, 57]. Studying the proteins involved in egg laying in monogeneans might be useful to better understand infection dynamics in the context of climate change.

## Competing interests

The authors declare that they have no competing interests.

## Supplementary material

The supplementary material of this article is available at https://www.parasitejournal.org/10.1051/parasite/2022052/olm.• *Supplementary file 1*:Detailed description of the functional annotation process, retrieval of lineage-specific GPCR, and code for the different pipelines used for assembly, annotation, read mapping and quantitative evaluation of the obtained transcriptome using BUSCO.• *Supplemental Figures S1–S8*:**– Supplementary Figure S1.**
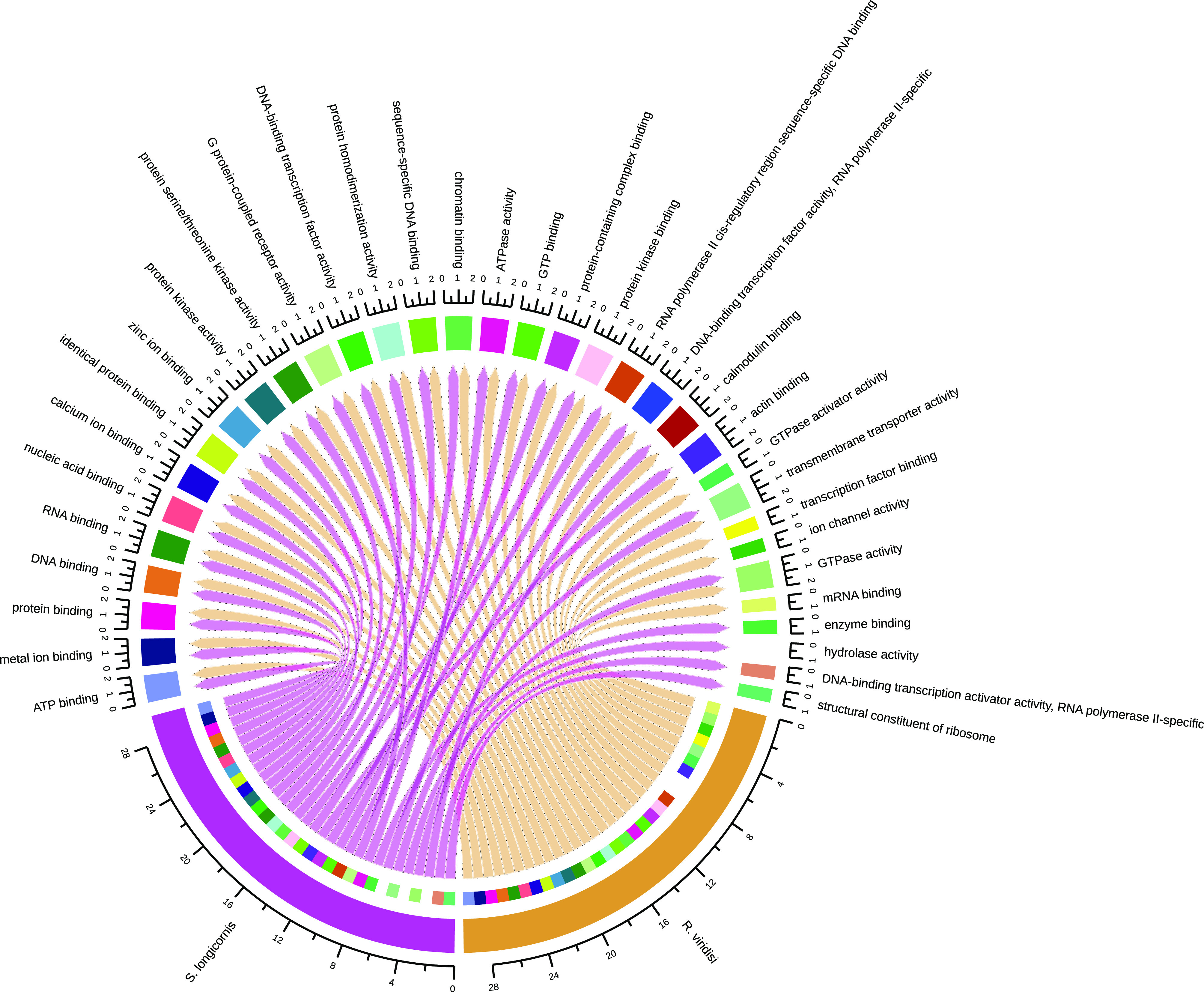
Assignment of Gene Ontology (GO) terms for the *Rhabdosynochus viridisi* and *Scutogyrus longicornis* ORFs. The number in the graph indicates the percent of ORFs related to *Molecular Function* terms.**– Supplementary Figure S2.**
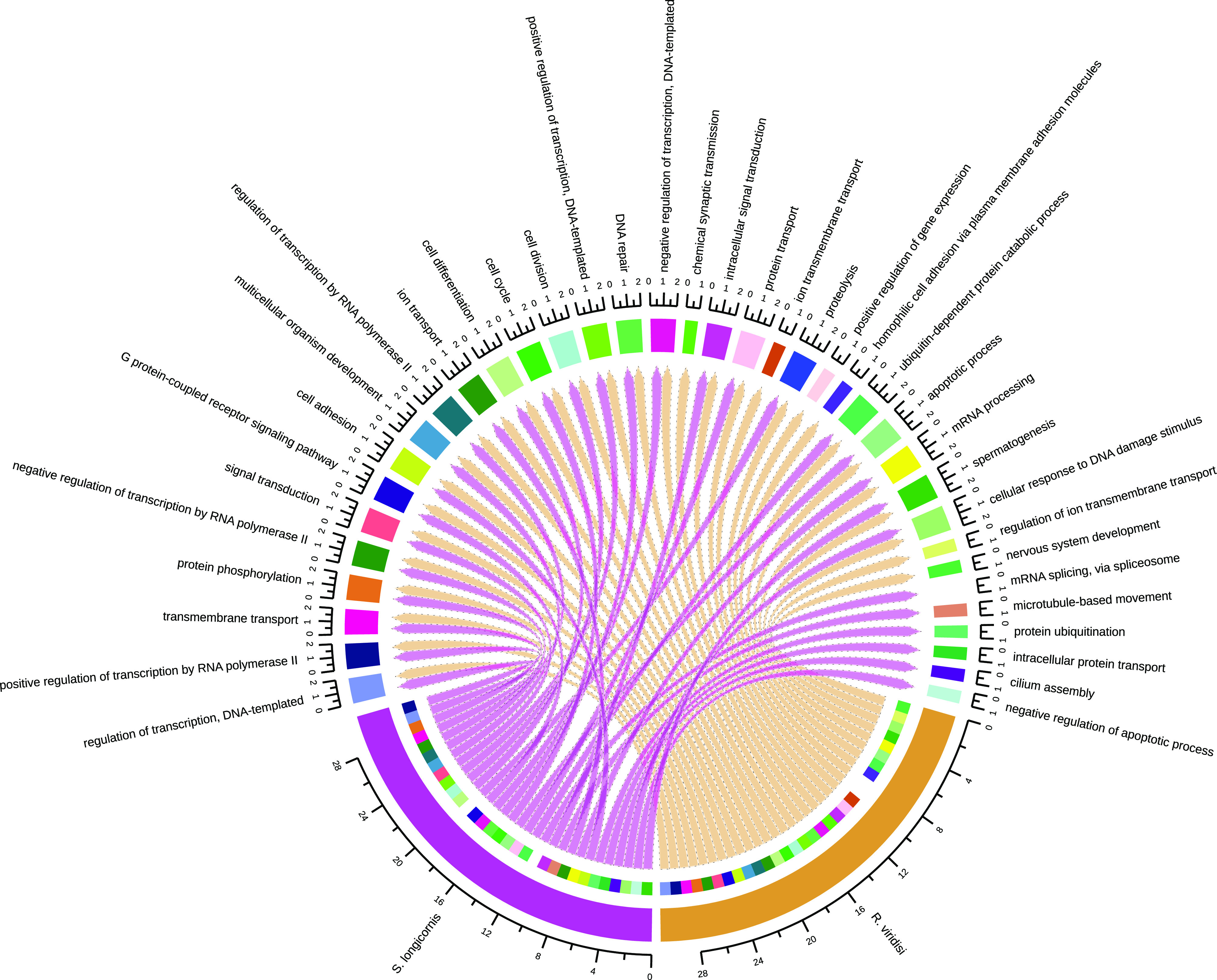
*Supplementary Figure S2*. Assignment of Gene Ontology (GO) terms for the *Rhabdosynochus viridisi* and *Scutogyrus longicornis* ORFs. The number in the graph indicates the percent of ORFs related to *Biological Process* terms.**– Supplementary Figure S3.**
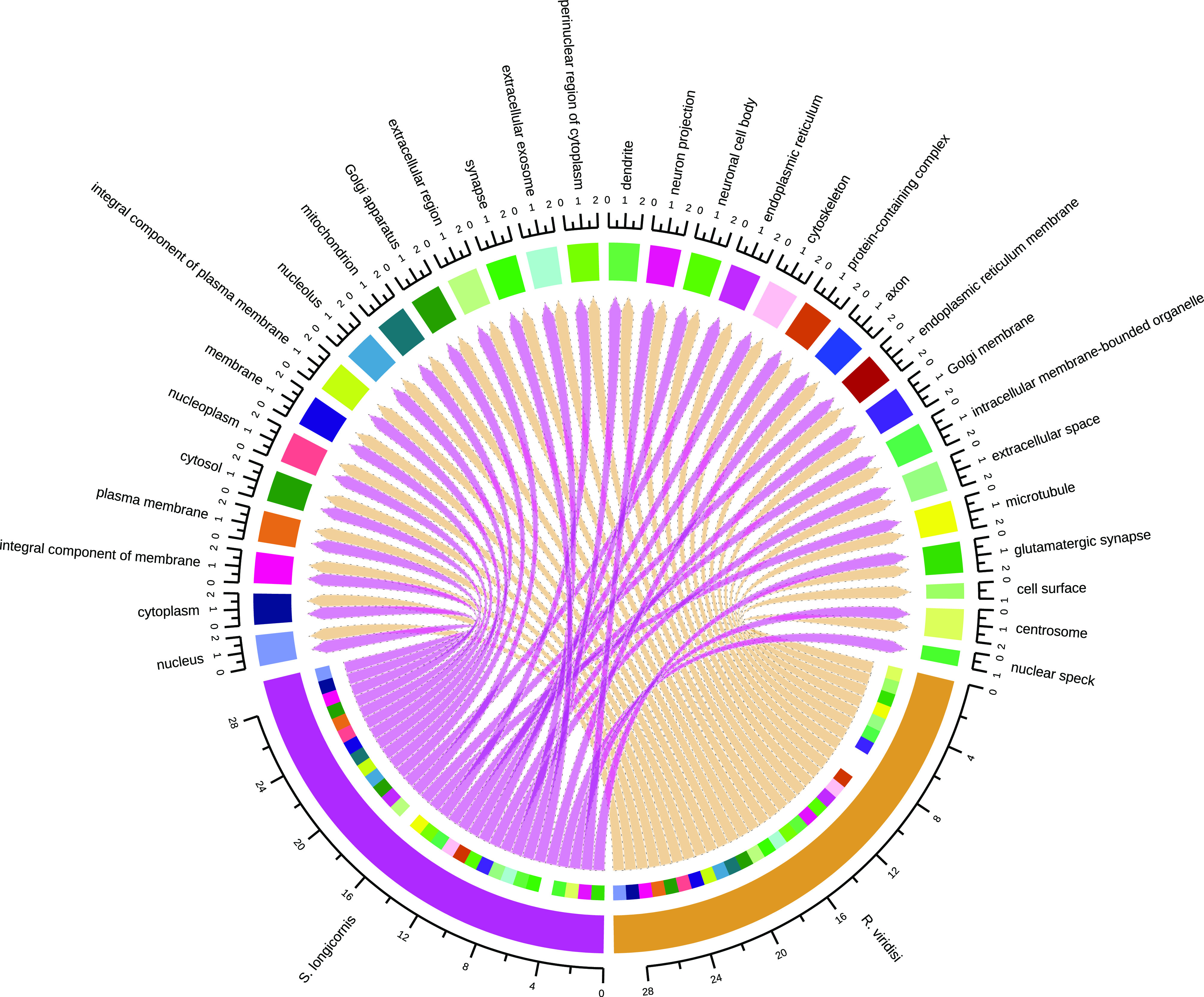
Assignment of Gene Ontology (GO) terms for the *Rhabdosynochus viridisi* and *Scutogyrus longicornis* ORFs. The number in the graph indicates the percent of ORFs related to *Cellular Component* terms.**– Supplementary Figure S4.**
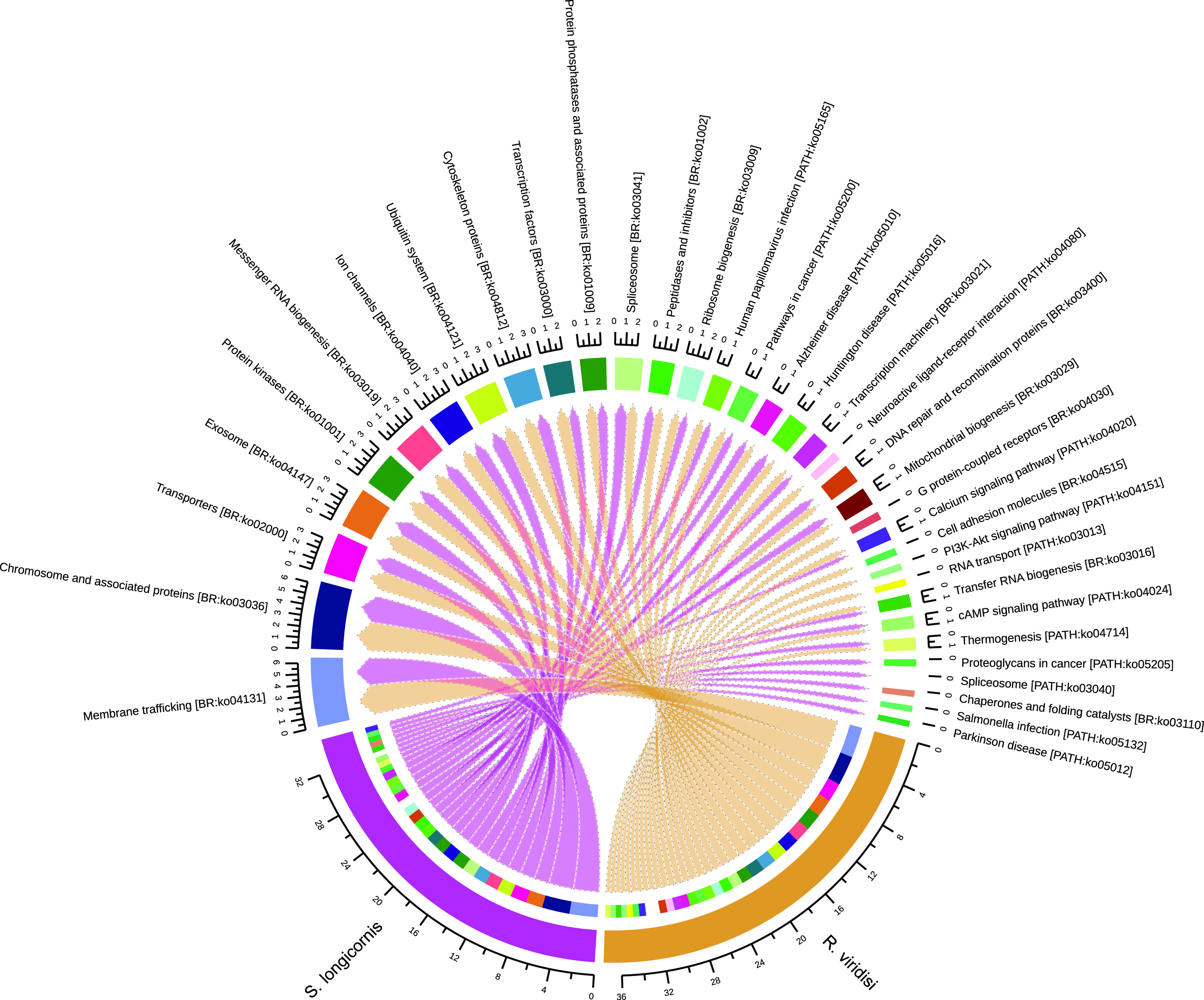
Top 30 most represented KEGG pathways in the *Rhabdosynochus viridisi* and *Scutogyrus longicornis* ORFs. The number in the graph indicates the percent of ORFs related to each term**– Supplementary Figure S5.**
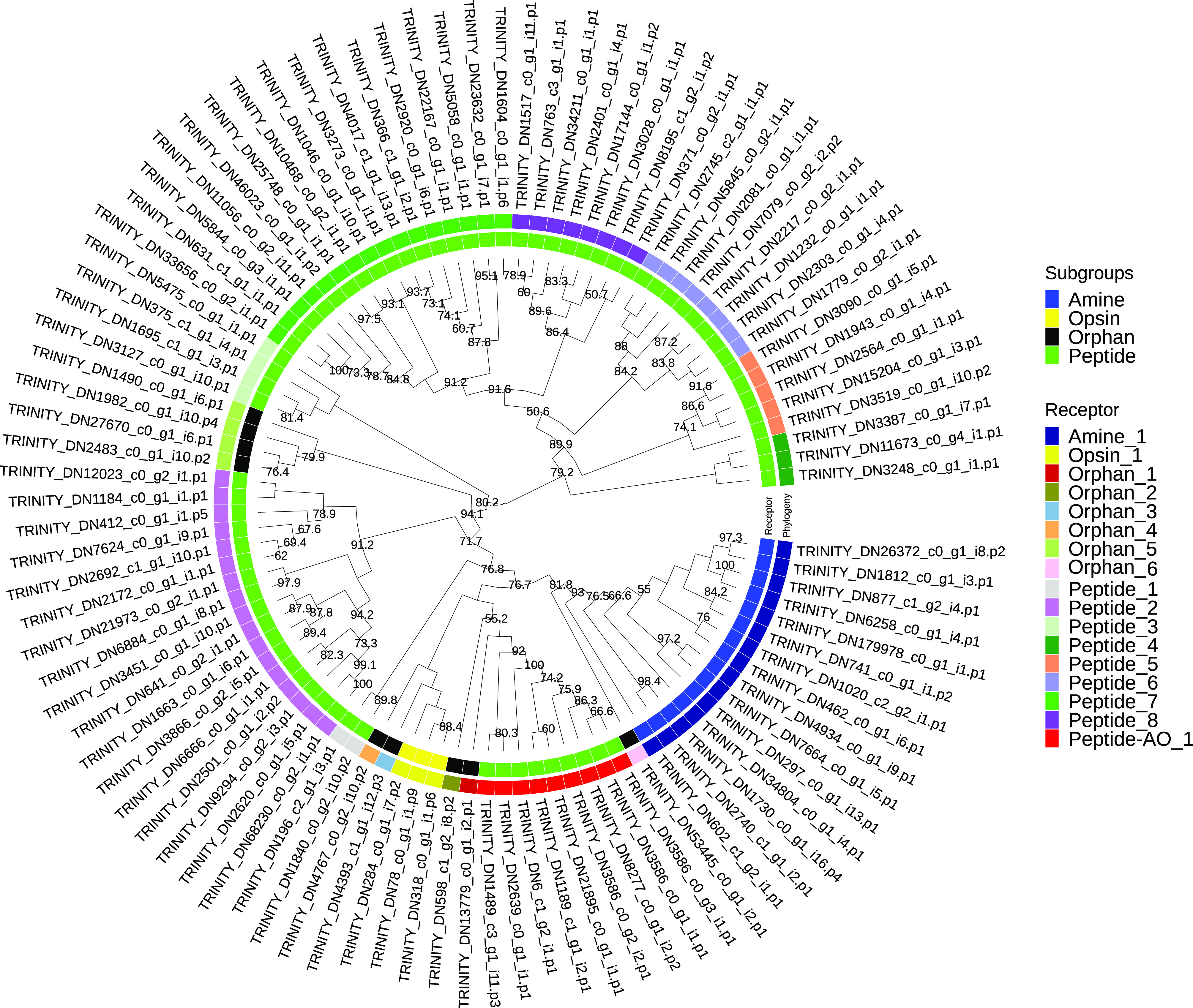
Phylogenetic classification of *Rhabdosynochus viridisi* rhodopsin-subfamily GPCRs. The midpoint-rooted phylogenetic tree was constructed using 1000 replicates of the approximate likelihood ratio test (similar to the Shimodaira-Hasegawa test). The LG+F+R6 model was implemented.**– Supplementary Figure S6.**
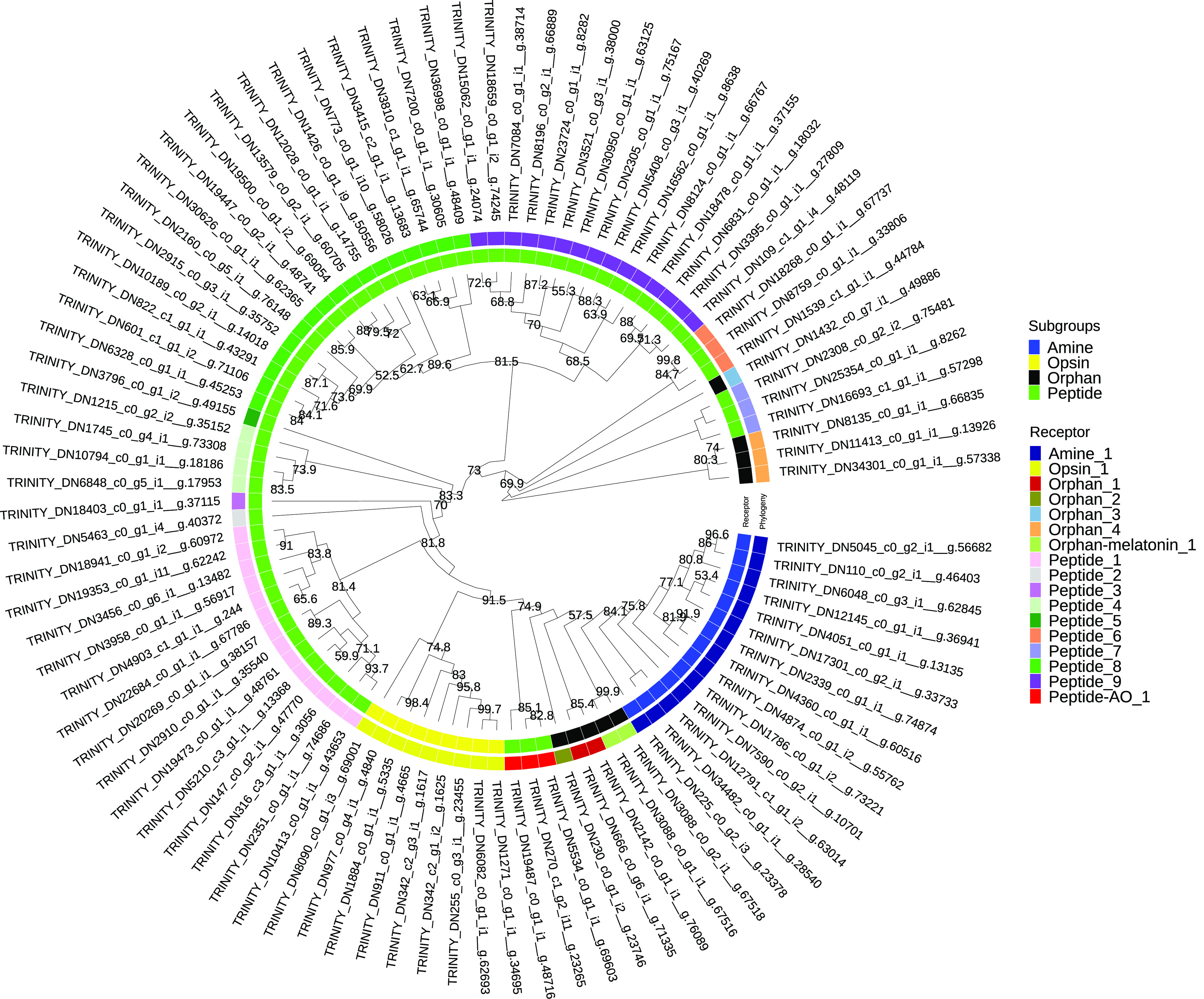
Phylogenetic classification of *Scutogyrus longicornis* GPCRs. The midpoint-rooted phylogenetic tree was constructed using 1000 replicates of the approximate likelihood ratio test (similar to the Shimodaira-Hasegawa test). The LG+F+G4 model was implemented.**– Supplementary Figure S7.**
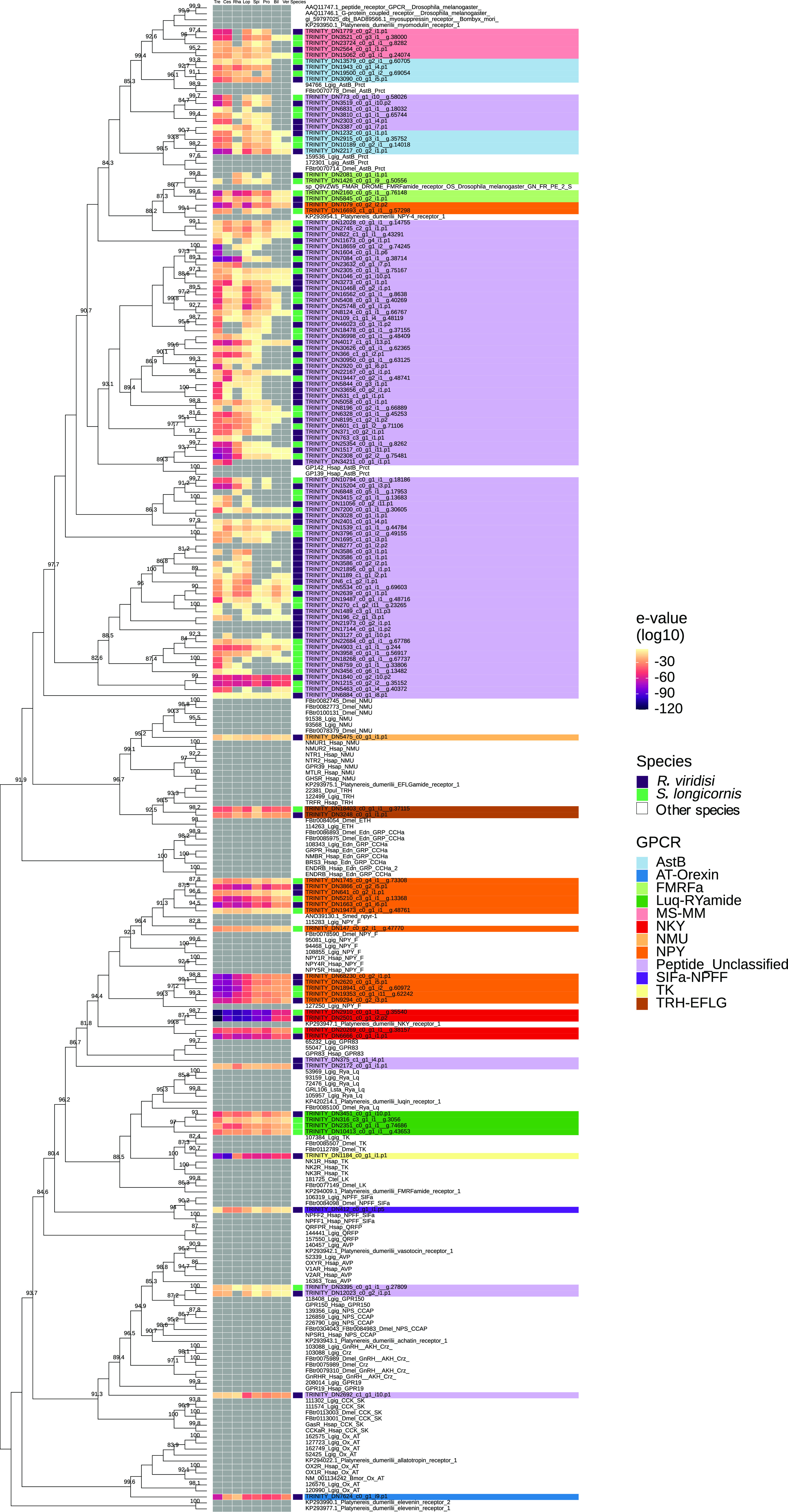
Phylogenetic analysis of *Rhabdosynochus viridisi* and *Scutogyrus longicornis* peptide-receptor GPCRs. The midpoint-rooted phylogenetic tree was constructed using 1000 replicates of the approximate likelihood ratio test (similar to the Shimodaira-Hasegawa test). The LG+F+R10 model was implemented. The colored boxes indicate the log10-transformed *e*-values obtained from the alignment of the *R. viridisi* and *S. longicornis* sequences against sequences of different taxa using the NCBI database (Tre, Trematoda; Ces, Cestoda; Rha, Rhabditophora; Lop, Lophotrochozoa; Spi, Spiralia; Pro, Protostomia; Bil, Bilateria; Ver, Vertebrata); the species to which the sequence belongs (*R. viridisi* or *S. longicornis*); and the type of receptor inferred from phylogenetic similarity to reference sequences.**– Supplementary Figure S8.**
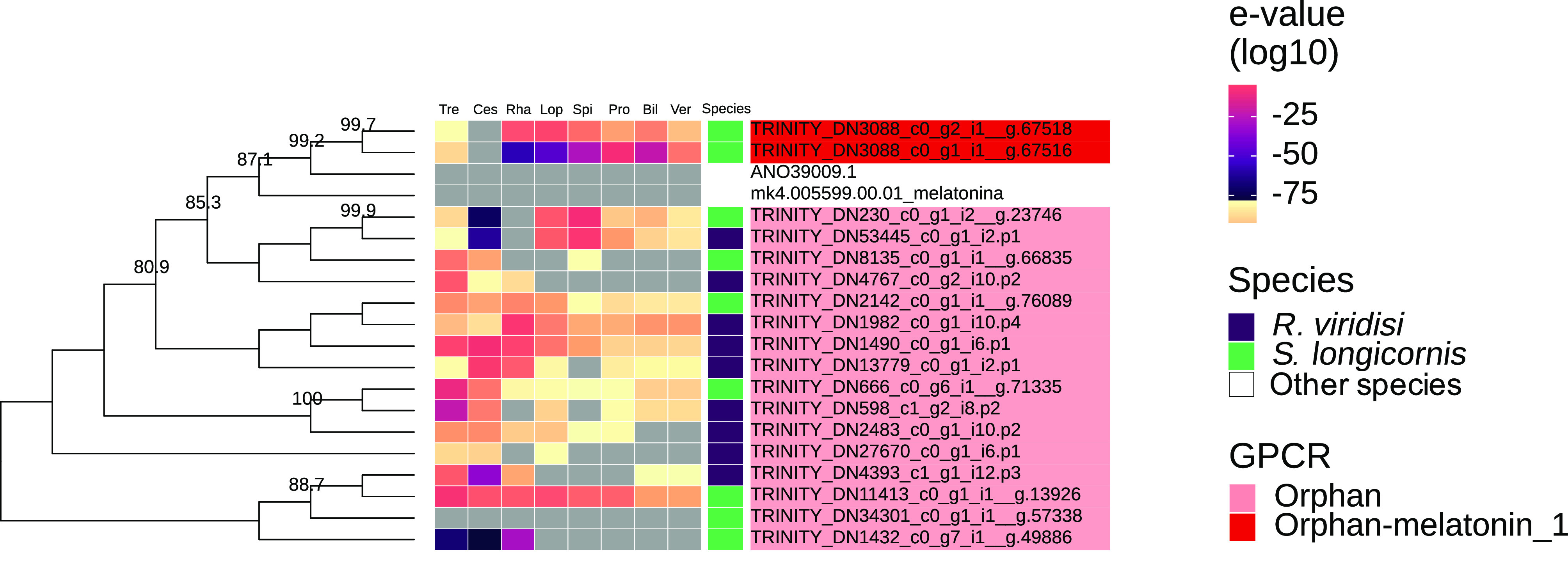
Phylogenetic analysis of *Rhabdosynochus viridisi* and *Scutogyrus longicornis* orphan GPCRs. The midpoint-rooted phylogenetic tree was constructed using 1000 replicates of the approximate likelihood ratio test (similar to the Shimodaira-Hasegawa test). The LG+F+R3 model was implemented. The colored boxes indicate the log10-transformed *e*-values obtained from the alignment of the *R. viridisi* and *S. longicornis* sequences against sequences of different taxa using the NCBI database (Tre, Trematoda; Ces, Cestoda; Rha, Rhabditophora; Lop, Lophotrochozoa; Spi, Spiralia; Pro, Protostomia; Bil, Bilateria; Ver, Vertebrata); the species to which the sequence belongs (*R. viridisi* or *S. longicornis*); and the type of receptor inferred from phylogenetic similarity to reference sequences.• *Supplemental Tables S1–S9*:– *Supplementary Table S1*.Annotation of putative proteins of *Rhabdosynochus viridisi*.– *Supplementary Table S2*.Annotation of putative proteins of *Scutogyrus longicornis*.– *Supplementary Table S3*. UniProt keyword annotation of putative proteins of *Rhabdosynochus viridisi* and *Scutogyrus longicornis.*– *Supplementary Table S4*. Information on each step of the classification of GPCRs of *Rhabdosynochus viridisi* and *Scutogyrus longicornis*.– *Supplementary Table S5*. Information on GPCR identification in each platyhelminth species.– *Supplementary Table S6*. Information on the *e*-values of the alignment of GPCRs.– *Supplementary Table S7*. UniProt annotation of putative proteins of *Rhabdosynochus viridisi* and *Scutogyrus longicornis.*– *Supplementary Table S8*. Domain annotation of putative proteins of *Rhabdosynochus viridisi* and *Scutogyrus longicornis.*– *Supplementary Table S9*. COG annotation of putative proteins of *Rhabdosynochus viridisi* and *Scutogyrus longicornis.*

## References

[1] Adell T, Martín-Durán JM, Saló E, Cebrià F. 2015. Platyhelminthes, Evolutionary Developmental Biology of Invertebrates. Springer: Vienna. p. 21–40.

[2] Aguirre-Fey D, Benítez-Villa GE, de Leon GPP, Rubio-Godoy M. 2015. Population dynamics of *Cichlidogyrus* spp. and *Scutogyrus* sp. (Monogenea) infecting farmed tilapia in Veracruz, México. Aquaculture, 443, 11–15.

[3] Andree KB, Roque A, Duncan N, Gisbert E, Estevez A, Tsertou MI, Katharios P. 2015. *Diplectanum sciaenae* (Van Beneden & Hesse, 1863) (Monogenea) infecting meagre, *Argyrosomus regius* (Asso, 1801) broodstock in Catalonia, Spain. Veterinary Parasitology: Regional Studies and Reports, 1, 75–79. 31018414 10.1016/j.vprsr.2016.02.006

[4] Apweiler R, Bairoch A, Wu CH, Barker WC, Boeckmann B, Ferro S, Gasteiger E, Huang H, Lopez R, Magrane M, Martin MJ, Natale DA, O’Donovan C, Redaschi N, Yeh LSL. 2004. UniProt: the universal protein knowledgebase. Nucleic Acids Research, 32(suppl_1), D115–D119. 14681372 10.1093/nar/gkh131 PMC308865

[5] Arensdorf AM, Marada S, Ogden SK. 2016. Smoothened regulation: a tale of two signals. Trends in Pharmacological Sciences, 37(1), 62–72. 26432668 10.1016/j.tips.2015.09.001 PMC4593303

[6] Ayers KL, Thérond PP. 2010. Evaluating Smoothened as a G-protein-coupled receptor for Hedgehog signaling. Trends in Cell Biology, 20(5), 287–298. 20207148 10.1016/j.tcb.2010.02.002

[7] Bahia D, Avelar L, Mortara RA, Khayath N, Yan Y, Noël C, Capron M, Dissous C, Pierce RJ, Oliveira G. 2006. SmPKC1, a new protein kinase C identified in the platyhelminth parasite *Schistosoma mansoni*. Biochemical and Biophysical Research Communications, 345(3), 1138–1148. 16713993 10.1016/j.bbrc.2006.05.025

[8] Bjarnadóttir TK, Fredriksson R, Schiöth HB. 2005. The gene repertoire and the common evolutionary history of glutamate, pheromone (V2R), taste (1) and other related G protein-coupled receptors. Gene, 362, 70–84. 16229975 10.1016/j.gene.2005.07.029

[9] Bockaert J, Fagni L, Dumuis A, Marin P. 2004. GPCR interacting proteins (GIP). Pharmacology & Therapeutics, 103(3), 203–221. 15464590 10.1016/j.pharmthera.2004.06.004

[10] Bolger AM, Lohse M, Usadel B. 2014. Trimmomatic: a flexible trimmer for Illumina sequence data. Bioinformatics, 30(15), 2114–2120. 24695404 10.1093/bioinformatics/btu170 PMC4103590

[11] Bryant DM, Johnson K, DiTommaso T, Tickle T, Couger MB, Payzin-Dogru D, Lee TJ, Leigh ND, Kuo TH, Davis FG, Bateman J, Bryant S, Guzikowski AR, Tsai SL, Coyne S, Ye WW, Freeman RM, Peshkin L, Tabin CJ, Regev A, Haas BJ, Whited JL. 2017. A tissue-mapped axolotl *de novo* transcriptome enables identification of limb regeneration factors. Cell Reports, 18(3), 762–776. 28099853 10.1016/j.celrep.2016.12.063 PMC5419050

[12] Buchfink B, Xie C, Huson DH. 2015. Fast and sensitive protein alignment using DIAMOND. Nature Methods, 12(1), 59–60. 25402007 10.1038/nmeth.3176

[13] Buchmann K, Roepstorff A, Waller PJ. 1992. Experimental selection of mebendazole-resistant gill monogeneans from the European eel, *Anguilla anguilla* L. Journal of Fish Diseases, 15(5), 393–408.

[14] Caffrey CR, Goupil L, Rebello KM, Dalton JP, Smith D. 2018. Cysteine proteases as digestive enzymes in parasitic helminths. PLoS Neglected Tropical Diseases, 12(8), e0005840. 30138310 10.1371/journal.pntd.0005840 PMC6107103

[15] Camacho C, Coulouris G, Avagyan V, Ma N, Papadopoulos J, Bealer K, Madden TL. 2009. BLAST+: architecture and applications. BMC Bioinformatics, 10(1), 1–9. 20003500 10.1186/1471-2105-10-421 PMC2803857

[16] Campos TD, Young ND, Korhonen PK, Hall RS, Mangiola S, Lonie A, Gasser RB. 2014. Identification of G protein-coupled receptors in *Schistosoma haematobium* and *S. mansoni* by comparative genomics. Parasites & Vectors, 7(1), 1–11. 24884876 10.1186/1756-3305-7-242 PMC4100253

[17] Caña-Bozada V, Llera-Herrera R, Fajer-Ávila EJ, Morales-Serna FN. 2021. Mitochondrial genome of *Rhabdosynochus viridisi* (Monogenea: Diplectanidae), a parasite of Pacific white snook *Centropomus viridis*. Journal of Helminthology, 95, e21. 33875027 10.1017/S0022149X21000146

[18] Caña-Bozada V, Llera-Herrera R, Fajer-Ávila EJ, Morales-Serna FN. 2021. Mitochondrial genome of *Scutogyrus longicornis* (Monogenea: Dactylogyridea), a parasite of Nile tilapia *Oreochromis niloticus*. Parasitology International, 81, 102281. 33401015 10.1016/j.parint.2020.102281

[19] Capella-Gutiérrez S, Silla-Martínez JM, Gabaldón T. 2009. trimAl: a tool for automated alignment trimming in large-scale phylogenetic analyses. Bioinformatics, 25(15), 1972–1973. 19505945 10.1093/bioinformatics/btp348 PMC2712344

[20] Chase DL, Koelle MR. 2007. Biogenic amine neurotransmitters in *C. elegans*. WormBook, 20, 1–15. 10.1895/wormbook.1.132.1 PMC4781333 18050501

[21] International Helminth Genomes Consortium. 2019. Comparative genomics of the major parasitic worms. Nature Genetics, 51(1), 163. 30397333 10.1038/s41588-018-0262-1 PMC6349046

[22] Cortés A, Mikeš L, Muñoz-Antolí C, Álvarez-Izquierdo M, Esteban JG, Horák P, Toledo R. 2019. Secreted cathepsin L-like peptidases are involved in the degradation of trapped antibodies on the surface of *Echinostoma caproni*. Parasitology Research, 118(12), 3377–3386. 31720841 10.1007/s00436-019-06487-4

[23] Cribb TH, Chisholm LA, Bray RA. 2002. Diversity in the Monogenea and Digenea: does lifestyle matter? International Journal for Parasitology, 32(3), 321–328. 11835972 10.1016/s0020-7519(01)00333-2

[24] Dahl G, Muller KJ. 2014. Innexin and pannexin channels and their signaling. FEBS Letters, 588(8), 1396–1402. 24632288 10.1016/j.febslet.2014.03.007

[25] Dezfuli BS, Giari L, Simoni E, Menegatti R, Shinn AP, Manera M. 2007. Gill histopathology of cultured European sea bass, *Dicentrarchus labrax* (L.), infected with *Diplectanum aequans* (Wagener 1857) Diesing 1958 (Diplectanidae: Monogenea). Parasitology Research, 100(4), 707–713. 17061113 10.1007/s00436-006-0343-4

[26] Dissous C, Grevelding CG, Long T. 2011. *Schistosoma mansoni* Polo-like kinases and their function in control of mitosis and parasite reproduction. Anais da Academia Brasileira de Ciências, 83, 627–635. 21670883 10.1590/s0001-37652011000200022

[27] Dissous C, Khayath N, Vicogne J, Capron M. 2006. Growth factor receptors in helminth parasites: signalling and host–parasite relationships. FEBS Letters, 580(12), 2968–2975. 16579990 10.1016/j.febslet.2006.03.046

[28] Donnelly S, O’Neill SM, Stack CM, Robinson MW, Turnbull L, Whitchurch C, Dalton JP. 2010. Helminth cysteine proteases inhibit TRIF-dependent activation of macrophages via degradation of TLR3. Journal of Biological Chemistry, 285(5), 3383–3392. 19923225 10.1074/jbc.M109.060368 PMC2823461

[29] Edgar RC. 2004. MUSCLE: multiple sequence alignment with high accuracy and high throughput. Bioinformatics, 32(5), 1792–1797. 10.1093/nar/gkh340 PMC390337 15034147

[30] Elphick MR, Mirabeau O, Larhammar D. 2018. Evolution of neuropeptide signaling systems. Journal of Experimental Biology, 221(3), jeb151092. 29440283 10.1242/jeb.151092 PMC5818035

[31] Fang H, Gough J. 2013. DcGO: database of domain-centric ontologies on functions, phenotypes, diseases and more. Nucleic Acids Research, 41(D1), D536–D544. 23161684 10.1093/nar/gks1080 PMC3531119

[32] Finn RD, Coggill P, Eberhardt RY, Eddy SR, Mistry J, Mitchell AL, Potter SC, Punta M, Qureshi M, Sangrador-Vegas A, Salazar GA, Bateman A. 2016. The Pfam protein families database: towards a more sustainable future. Nucleic Acids Research, 44(D1), D279–D285. 26673716 10.1093/nar/gkv1344 PMC4702930

[33] Fredriksson R, Lagerström MC, Lundin LG, Schiöth HB. 2003. The G-protein-coupled receptors in the human genome form five main families. Phylogenetic analysis, paralogon groups, and fingerprints. Molecular Pharmacology, 63(6), 1256–1272. 12761335 10.1124/mol.63.6.1256

[34] Fu L, Niu B, Zhu Z, Wu S, Li W. 2012. CD-HIT: accelerated for clustering the next-generation sequencing data. Bioinformatics, 28(23), 3150–3152. 23060610 10.1093/bioinformatics/bts565 PMC3516142

[35] Gene Ontology Consortium. 2008. The gene ontology project in 2008. Nucleic Acids Research, 36(suppl_1), D440–D444. 17984083 10.1093/nar/gkm883 PMC2238979

[36] Giuliani S, Silva AC, Borba JV, Ramos PI, Paveley RA, Muratov EN, Andrade CH, Furnham N. 2018. Computationally-guided drug repurposing enables the discovery of kinase targets and inhibitors as new schistosomicidal agents. PLoS Computational Biology, 14(10), e1006515. 30346968 10.1371/journal.pcbi.1006515 PMC6211772

[37] Grabherr MG, Haas BJ, Yassour M, Levin JZ, Thompson DA, Amit I, Adiconis X, Fan L, Raychowdhury R, Zeng Q, Chen Z, Mauceli E, Hacohen N, Gnirke A, Rhind N, di Palma F, Birren BW, Nusbaum C, Lindblad-Toh K, Friedman N, Regev A. 2011. Full-length transcriptome assembly from RNA-Seq data without a reference genome. Nature Biotechnology, 29(7), 644–652. 10.1038/nbt.1883 PMC3571712 21572440

[38] Guidi A, Mansour NR, Paveley RA, Carruthers IM, Besnard J, Hopkins AL, Gilbert IH, Bickle QD. 2015. Application of RNAi to genomic drug target validation in schistosomes. PLoS Neglected Tropical Diseases, 5, e0003801. 10.1371/journal.pntd.0003801 PMC4438872 25992548

[39] Güiza J, Barría I, Sáez JC, Vega JL. 2018. Innexins: expression, regulation, and functions. Frontiers in Physiology, 9, 1414. 30364195 10.3389/fphys.2018.01414 PMC6193117

[40] Haas BJ, Papanicolaou A, Yassour M, Grabherr M, Blood PD, Bowden J, Couger MB, Eccles D, Li B, Lieber M, MacManes MD, Ott M, Orvis J, Pochet N, Strozzi F, Weeks N, Westerman R, William T, Dewey CN, Henschel R, LeDuc RD, Friedman N, Regev A. 2013. *De novo* transcript sequence reconstruction from RNA-seq using the Trinity platform for reference generation and analysis. Nature Protocols, 8(8), 1494–1512. 23845962 10.1038/nprot.2013.084 PMC3875132

[41] Hahn C, Fromm B, Bachmann L. 2014. Comparative genomics of flatworms (Platyhelminthes) reveals shared genomic features of ecto-and endoparastic neodermata. Genome Biology and Evolution, 6(5), 1105–1117. 24732282 10.1093/gbe/evu078 PMC4040987

[42] Hauser AS, Attwood MM, Rask-Andersen M, Schiöth HB, Gloriam DE. 2017. Trends in GPCR drug discovery: new agents, targets and indications. Nature Reviews Drug Discovery, 16(12), 829–842. 29075003 10.1038/nrd.2017.178 PMC6882681

[43] Hoai TD. 2020. Reproductive strategies of parasitic flatworms (Platyhelminthes, Monogenea): the impact on parasite management in aquaculture. Aquaculture International, 28(1), 421–447.

[44] Hoai TD, Hutson KS. 2014. Reproductive strategies of the insidious fish ectoparasite, *Neobenedenia* sp. (Capsalidae: Monogenea). PLoS One, 9(9), e108801. 25264931 10.1371/journal.pone.0108801 PMC4181869

[45] Houhou H, Puckelwaldt O, Strube C, Haeberlein S. 2019. Reference gene analysis and its use for kinase expression profiling in *Fasciola hepatica*. Scientific Reports, 9(1), 1–14. 31676853 10.1038/s41598-019-52416-x PMC6825121

[46] Howe KL, Bolt BJ, Shafie M, Kersey P, Berriman M. 2017. WormBase ParaSite – a comprehensive resource for helminth genomics. Molecular and Biochemical Parasitology, 215, 2–10. 27899279 10.1016/j.molbiopara.2016.11.005 PMC5486357

[47] Huang HC, Klein PS. 2004. The Frizzled family: receptors for multiple signal transduction pathways. Genome Biology, 5(7), 1–7. 10.1186/gb-2004-5-7-234 PMC463283 15239825

[48] Hübner CA, Jentsch TJ. 2002. Ion channel diseases. Human Molecular Genetics, 11(20), 2435–2445. 12351579 10.1093/hmg/11.20.2435

[49] Huerta-Cepas J, Szklarczyk D, Forslund K, Cook H, Heller D, Walter MC, Rattei T, Mende DR, Sunagawa S, Kuhn M, Jensen LJ, von Mering C, Bork P. 2016. eggNOG 4.5: a hierarchical orthology framework with improved functional annotations for eukaryotic, prokaryotic and viral sequences. Nucleic Acids Research, 44(D1), D286–D293. 26582926 10.1093/nar/gkv1248 PMC4702882

[50] James CE, Hudson AL, Davey MW. 2009. Drug resistance mechanisms in helminths: Is it survival of the fittest? Trends in Parasitology, 25(7), 328–335. 19541539 10.1016/j.pt.2009.04.004

[51] Jedličková L, Dvořáková H, Dvořák J, Kašný M, Ulrychova L, Vorel J, Žárský V, Mikeš L. 2018. Cysteine peptidases of *Eudiplozoon nipponicum*: a broad repertoire of structurally assorted cathepsins L in contrast to the scarcity of cathepsins B in an invasive species of haematophagous monogenean of common carp. Parasites & Vectors, 11(1), 1–17. 29510760 10.1186/s13071-018-2666-2 PMC5840727

[52] Jerônimo GT, Speck GM, Cechinel MM, Gonçalves ELT, Martins ML. 2011. Seasonal variation on the ectoparasitic communities of Nile tilapia cultured in three regions in southern Brazil. Brazilian Journal of Biology, 71, 365–373. 10.1590/s1519-69842011000300005 21755153

[53] Kalyaanamoorthy S, Minh BQ, Wong TK, Von Haeseler A, Jermiin LS. 2017. ModelFinder: fast model selection for accurate phylogenetic estimates. Nature Methods, 14(6), 587–589. 28481363 10.1038/nmeth.4285 PMC5453245

[54] Kanehisa M, Goto S, Sato Y, Furumichi M, Tanabe M. 2012. KEGG for integration and interpretation of large-scale molecular data sets. Nucleic Acids Research, 40(D1), D109–D114. 22080510 10.1093/nar/gkr988 PMC3245020

[55] Katoh K, Standley DM. 2013. MAFFT multiple sequence alignment software version 7: improvements in performance and usability. Molecular Biology and Evolution, 30(4), 772–780. 23329690 10.1093/molbev/mst010 PMC3603318

[56] Kearn GC. 1967. Experiments on host-finding and host-specificity in the monogenean skin parasite *Entobdella soleae*. Parasitology, 57(3), 585–605. 6069119 10.1017/s0031182000072450

[57] Kearn GC. 1986. The eggs of monogeneans. Advances in Parasitology, 25, 175–273. 3535435 10.1016/s0065-308x(08)60344-9

[58] Kearn GC, Macdonald S. 1976. The chemical nature of host hatching factors in the monogenean skin parasites *Entobdella soleae* and *Acanthocotyle lobianchi*. International Journal for Parasitology, 6(6), 457–466. 1010670 10.1016/0020-7519(76)90082-5

[59] Keating CD, Kriek N, Daniels M, Ashcroft NR, Hopper NA, Siney EJ, Siney EJ, Burke JF. 2003. Whole-genome analysis of 60 G protein-coupled receptors in *Caenorhabditis elegans* by gene knockout with RNAi. Current Biology, 13(19), 1715–1720. 14521838 10.1016/j.cub.2003.09.003

[60] Kerfeld CA, Scott KM. 2011. Using BLAST to teach “E-value-tionary” concepts. PLoS Biology, 9(2), e1001014. 21304918 10.1371/journal.pbio.1001014 PMC3032543

[61] Konczal M, Przesmycka KJ, Mohammed RS, Phillips KP, Camara F, Chmielewski S, Hahn C, Guigo R, Cable J, Radwan J. 2020. Gene duplications, divergence and recombination shape adaptive evolution of the fish ectoparasite *Gyrodactylus bullatarudis*. Molecular Ecology, 29(8), 1494–1507. 32222008 10.1111/mec.15421

[62] Koziol U, Koziol M, Preza M, Costábile A, Brehm K, Castillo E. 2016. *De novo* discovery of neuropeptides in the genomes of parasitic flatworms using a novel comparative approach. International Journal for Parasitology, 46(11), 709–721. 27388856 10.1016/j.ijpara.2016.05.007

[63] Krogh A, Larsson B, Von Heijne G, Sonnhammer EL. 2001. Predicting transmembrane protein topology with a hidden Markov model: application to complete genomes. Journal of Molecular Biology, 305(3), 567–580. 11152613 10.1006/jmbi.2000.4315

[64] Kumkate S, Chunchob S, Janvilisri T. 2008. Expression of ATP-binding cassette multidrug transporters in the giant liver fluke *Fasciola gigantica* and their possible involvement in the transport of bile salts and anthelmintics. Molecular and Cellular Biochemistry, 317(1), 77–84. 18543082 10.1007/s11010-008-9833-2

[65] Langenhan T, Aust G, Hamann J. 2013. Sticky signaling – Adhesion class G protein–coupled receptors take the stage. Science Signaling, 6(276), re3. 23695165 10.1126/scisignal.2003825

[66] Langmead B, Salzberg SL. 2012. Fast gapped-read alignment with Bowtie 2. Nature Methods, 9(4), 357–359. 22388286 10.1038/nmeth.1923 PMC3322381

[67] Laumer CE, Hejnol A, Giribet G. 2015. Nuclear genomic signals of the ‘microturbellarian’ roots of platyhelminth evolutionary innovation. eLife, 4, e05503. 10.7554/eLife.05503 PMC4398949 25764302

[68] Lee HG, Seong CS, Kim YC, Davis RL, Han KA. 2003. Octopamine receptor OAMB is required for ovulation in *Drosophila melanogaster*. Developmental Biology, 264(1), 179–190. 14623240 10.1016/j.ydbio.2003.07.018

[69] Lee IH, Procko C, Lu Y, Shaham S. 2021. Stress-Induced neural plasticity mediated by glial GPCR REMO-1 promotes *C. elegans* adaptive behavior. Cell Reports, 34(2), 108607. 33440160 10.1016/j.celrep.2020.108607 PMC7845533

[70] Li H, Handsaker B, Wysoker A, Fennell T, Ruan J, Homer N, Marth G, Abecasis G, Durbin R. 2009. The sequence alignment/map format and SAMtools. Bioinformatics, 25(16), 2078–2079. 19505943 10.1093/bioinformatics/btp352 PMC2723002

[71] Liang D, Zhao M, Wang T, McManus DP, Cummins SF. 2016. GPCR and IR genes in *Schistosoma mansoni* miracidia. Parasites & Vectors, 9(1), 1–12. 27784323 10.1186/s13071-016-1837-2 PMC5080760

[72] Lim J, Sabandal PR, Fernandez A, Sabandal JM, Lee HG, Evans P, Han KA. 2014. The octopamine receptor Octβ2R regulates ovulation in *Drosophila melanogaster*. PloS One, 9(8), e104441. 25099506 10.1371/journal.pone.0104441 PMC4123956

[73] Lok JB. 2016. Signaling in parasitic nematodes: physicochemical communication between host and parasite and endogenous molecular transduction pathways governing worm development and survival. Current Clinical Microbiology Reports, 3(4), 186–197. 28781934 10.1007/s40588-016-0046-2 PMC5543980

[74] MacDonald K, Kimber MJ, Day TA, Ribeiro P. 2015. A constitutively active G protein-coupled acetylcholine receptor regulates motility of larval *Schistosoma mansoni*. Molecular and Biochemical Parasitology, 202(1), 29–37. 10.1016/j.molbiopara.2015.09.001 PMC4607267 26365538

[75] MacDonald S, Jones A. 1978. Egg-laying and hatching rhythms in the monogenean *Diplozoon homoion gracile* from the southern barbel (*Barbus meridionalis*). Journal of Helminthology, 52(1), 23–28. 659823 10.1017/s0022149x00005071

[76] Manglik A, Kobilka B. 2014. The role of protein dynamics in GPCR function: insights from the β2AR and rhodopsin. Current Opinion in Cell Biology, 27, 136–143. 24534489 10.1016/j.ceb.2014.01.008 PMC3986065

[77] Manning G, Whyte DB, Martinez R, Hunter T, Sudarsanam S. 2002. The protein kinase complement of the human genome. Science, 298(5600), 1912–1934. 12471243 10.1126/science.1075762

[78] Marks NJ, Maule AG. 2010. Neuropeptides in helminths: occurrence and distribution, in Neuropeptide Systems as Targets for Parasite and Pest Control. Advances in Experimental Medicine and Biology, Vol. 692. Geary TG, Maule AG, Editors. Springer: New York, NY. p. 49–77.

[79] McVeigh P. 2020. Post-genomic progress in helminth parasitology. Parasitology, 147(8), 835–840. 32252832 10.1017/S0031182020000591 PMC7284816

[80] McVeigh P, Kimber MJ, Novozhilova E, Day TA. 2005. Neuropeptide signaling systems in flatworms. Parasitology, 131(S1), S41–S55. 16569292 10.1017/S0031182005008851

[81] McVeigh P, McCusker P, Robb E, Wells D, Gardiner E, Mousley A, Marks NJ, Maule AG. 2018. Reasons to be nervous about flukicide discovery. Trends in Parasitology, 34(3), 184–196. 29269027 10.1016/j.pt.2017.11.010

[82] McVeigh P, McCammick E, McCusker P, Wells D, Hodgkinson J, Paterson S, Mousleya A, Marks NJ, Maule AG. 2018. Profiling G protein-coupled receptors of *Fasciola hepatica* identifies orphan rhodopsins unique to phylum Platyhelminthes. International Journal for Parasitology: Drugs and Drug Resistance, 8(1), 87–103. 29474932 10.1016/j.ijpddr.2018.01.001 PMC6114109

[83] Montero-Mendieta S, Grabherr M, Lantz H, De la Riva I, Leonard JA, Webster MT, Vilà C. 2017. A practical guide to build *de-novo* assemblies for single tissues of non-model organisms: the example of a Neotropical frog. PeerJ, 5, e3702. 28879061 10.7717/peerj.3702 PMC5582611

[84] Morales-Serna FN, López-Moreno DG, Medina-Guerrero RM, Abad-Rosales SM, Martínez-Brown JM, Ibarra-Castro L, Fajer-Avila EJ. 2020. Toxicity of formalin for juvenile *Centropomus viridis* and in vitro efficacy against the parasite *Rhabdosynochus* sp. (Monogenea: Diplectanidae). Journal of Applied Ichthyology, 36(5), 740–744.

[85] Morita M, Hall F, Best JB, Gern W. 1987. Photoperiodic modulation of cephalic melatonin in planarians. Journal of Experimental Zoology, 241(3), 383–388. 3585272 10.1002/jez.1402410314

[86] Nguyen LT, Schmidt HA, Von Haeseler A, Minh BQ. 2015. IQ-TREE: a fast and effective stochastic algorithm for estimating maximum-likelihood phylogenies. Molecular Biology and Evolution, 32(1), 268–274. 25371430 10.1093/molbev/msu300 PMC4271533

[87] Nordström KJ, Lagerström MC, Wallér LM, Fredriksson R, Schiöth HB. 2009. The Secretin GPCRs descended from the family of Adhesion GPCRs. Molecular Biology and Evolution, 26(1), 71–84. 18845549 10.1093/molbev/msn228

[88] Ogawa K. 2015. Diseases of cultured marine fishes caused by Platyhelminthes (Monogenea, Digenea, Cestoda). Parasitology, 142(1), 178–195. 24998438 10.1017/S0031182014000808

[89] Overington JP, Al-Lazikani B, Hopkins AL. 2006. How many drug targets are there? Nature Reviews Drug Discovery, 5(12), 993–996. 17139284 10.1038/nrd2199

[90] Oviedo NJ, Morokuma J, Walentek P, Kema IP, Gu MB, Ahn JM, Hwang JS, Gojobori T, Levin M. 2010. Long-range neural and gap junction protein-mediated cues control polarity during planarian regeneration. Developmental Biology, 339(1), 188–199. 20026026 10.1016/j.ydbio.2009.12.012 PMC2823934

[91] Patocka N, Sharma N, Rashid M, Ribeiro P. 2014. Serotonin signaling in *Schistosoma mansoni*: A Serotonin–activated G protein-coupled receptor controls parasite movement. PLoS Pathogens, 10(1), e1003878. 24453972 10.1371/journal.ppat.1003878 PMC3894222

[92] Petersen TN, Brunak S, Von Heijne G, Nielsen H. 2011. SignalP 4.0: discriminating signal peptides from transmembrane regions. Nature Methods, 8(10), 785–786. 21959131 10.1038/nmeth.1701

[93] Rao YZ, Yang TB. 2007. cDNA cloning, mRNA expression and recombinant expression of a cathepsin L-like cysteine protease from *Neobenedenia melleni* (Monogenea: Capsalidae). Aquaculture., 269(1–4), 41–53.

[94] Ribeiro P, Geary TG. 2010. Neuronal signaling in schistosomes: current status and prospects for postgenomics. Canadian Journal of Zoology, 88(1), 1–22.

[95] Robinson MW, Tort JF, Lowther J, Donnelly SM, Wong E, Xu W, Stack CM, Padula M, Herbert B, Dalton JP. 2008. Proteomics and phylogenetic analysis of the cathepsin L protease family of the helminth pathogen *Fasciola hepatica*: expansion of a repertoire of virulence-associated factors. Molecular and Cellular Proteomics, 7(6), 1111–1123. 18296439 10.1074/mcp.M700560-MCP200

[96] Saberi A, Jamal A, Beets I, Schoofs L, Newmark PA. 2016. GPCRs direct germline development and somatic gonad function in planarians. PLoS Biolpgy, 14(5), e1002457. 10.1371/journal.pbio.1002457 PMC4862687 27163480

[97] Santos BM, Dias BK, Nakabashi M, Garcia CR. 2021. The knockout for G Protein-Coupled Receptor-Like PfSR25 increases the susceptibility of malaria parasites to the antimalarials lumefantrine and piperaquine but not to medicine for malaria venture compounds. Frontiers in Microbiology, 12, 638869. 33790879 10.3389/fmicb.2021.638869 PMC8006397

[98] Seppey M, Manni M, Zdobnov EM. 2019. BUSCO: assessing genome assembly and annotation completeness. Gene Prediction, 1962, 227–245. 10.1007/978-1-4939-9173-0_14 31020564

[99] Stoltzfus JD, Minot S, Berriman M, Nolan TJ, Lok JB. 2012. RNAseq analysis of the parasitic nematode *Strongyloides stercoralis* reveals divergent regulation of canonical dauer pathways. PLoS Neglected Tropical Diseases, 6(10), e1854. 23145190 10.1371/journal.pntd.0001854 PMC3493385

[100] Taman A, Ribeiro P. 2009. Investigation of a dopamine receptor in *Schistosoma mansoni*: functional studies and immunolocalization. Molecular and Biochemical Parasitology, 168(1), 24–33. 19545592 10.1016/j.molbiopara.2009.06.003

[101] Tatusov RL, Galperin MY, Natale DA, Koonin EV. 2000. The COG database: a tool for genome-scale analysis of protein functions and evolution. Nucleic Acids Research, 28(1), 33–36. 10592175 10.1093/nar/28.1.33 PMC102395

[102] Van Bel M, Proost S, Van Neste C, Deforce D, Van de Peer Y, Vandepoele K. 2013. TRAPID: an efficient online tool for the functional and comparative analysis of *de novo* RNA-Seq transcriptomes. Genome Biology, 14(12), 1–10. 10.1186/gb-2013-14-12-r134 PMC4053847 24330842

[103] Vega JL, Subiabre M, Figueroa F, Schalper KA, Osorio L, González J, Sáez JC. 2013. Role of gap junctions and hemichannels in parasitic infections. BioMed Research International, 2013, 589130. 24236292 10.1155/2013/589130 PMC3819887

[104] Vorel J, Cwiklinski K, Roudnický P, Ilgová J, Jedličková L, Dalton JP, Mikeš L, Gelnar M, Kašný M. 2021. *Eudiplozoon nipponicum* (Monogenea, Diplozoidae) and its adaptation to haematophagy as revealed by transcriptome and secretome profiling. BMC Genomics, 22(1), 1–17. 33858339 10.1186/s12864-021-07589-z PMC8050918

[105] Watanabe M. 2017. Gap junction in the teleost fish lineage: duplicated connexins may contribute to skin pattern formation and body shape determination. Frontiers in Cell and Development Biology, 5, 13. 10.3389/fcell.2017.00013 PMC5318405 28271062

[106] Waterhouse RM, Seppey M, Simão FA, Manni M, Ioannidis P, Klioutchnikov G, Kriventseva EV, Zdobnov EM. 2018. BUSCO applications from quality assessments to gene prediction and phylogenomics. Molecular Biology and Evolution, 35(3), 543–548. 29220515 10.1093/molbev/msx319 PMC5850278

[107] Wolstenholme AJ. 2011. Ion channels and receptor as targets for the control of parasitic nematodes. International Journal for Parasitology: Drugs and Drug Resistance, 1(1), 2–13. 24533259 10.1016/j.ijpddr.2011.09.003 PMC3898135

[108] Wolstenholme AJ, Fairweather I, Prichard R, von Samson-Himmelstjerna G, Sangster NC. 2004. Drug resistance in veterinary helminths. Trends in Parasitology, 20(10), 469–476. 15363440 10.1016/j.pt.2004.07.010

[109] Yermakova ON, Yermakov AM, Tiras KP, Lednev VV. 2009. Melatonin effect on the regeneration of the flatworm *Girardia tigrina*. Russian Journal of Developmental Biology, 40(6), 382–385. 20058790

[110] Yu G, Smith DK, Zhu H, Guan Y, Lam TTY. 2017. ggtree: an R package for visualization and annotation of phylogenetic trees with their covariates and other associated data. Methods in Ecology and Evolution, 8(1), 28–36.

[111] Zamanian M, Kimber MJ, McVeigh P, Carlson SA, Maule AG, Day TA. 2011. The repertoire of G protein-coupled receptors in the human parasite *Schistosoma mansoni* and the model organism *Schmidtea mediterranea*. BMC Genomics, 12(1), 1–21. 10.1186/1471-2164-12-596 PMC3261222 22145649

[112] Zhang S, Zhi T, Xu X, Zheng Y, Bilong CFB, Pariselle A, Yang T. 2019. Monogenean fauna of alien tilapias (Cichlidae) in south China. Parasite, 26. 10.1051/parasite/2019003 PMC6361074 30714897

[113] Zhang Z, Wu J, Yu J, Xiao J. 2013. A brief review on the evolution of GPCR: conservation and diversification. Open Journal of Genetics, 2(04), 11.

