# Supplementary material for: De novo transcriptome assembly and identification of G-Protein-Coupled-Receptors (GPCRs) in two species of monogenean parasites of fish
Source: Parasite. 2022 Nov 9;29:51. doi: 10.1051/parasite/2022052 (PMC9645230; doi:10.1051/parasite/2022052)
Supplement: Supplementary file 1 — • Supplementary file 1: Detailed description of the functional annotation process, retrieval of lineage-specific GPCR, and code for the different pipelines used for assembly, annotation, read mapping and quantitative evaluation of the obtained transcriptome using BUSCO. [file parasite-29-51-s1.pdf]

SUPPLEMENTARY FILE

De novo transcriptome assembly and identification of GPCRs in two species of monogenean parasites of fish

Authors

Víctor Caña-Bozada, F. Neptalí Morales-Serna, Emma J. Fajer-Ávila, Raúl Llera-Herrera

Affiliations

Centro de Investigación en Alimentación y Desarrollo, A.C. Mazatlán, Sinaloa, Mexico

Instituto de Ciencias del Mar y Limnología, Universidad Nacional Autónoma de México, Mazatlán, Sinaloa, Mexico

Functional annotation

Although the annotation was done for all the putative proteins, we present results of the longest, not redundant proteins to avoid overrepresentation of sequences. After removing redundant sequences (isoforms), the representative proteins were reduced from 47,187 to 23,857 in *R. viridisi*, and from 25,696 to 12,020 in *S. longicornis.* For *R. viridisi*, 3214, 2761, 2798, 2114, and 1205 protein sequences were aligned to the Swiss-Prot, Pfam, GO, KEGG, and COG databases, respectively. In addition, 6824 proteins were predicted with TM domain and 2823 with a signal peptide. For *S. longicornis*, 5849, 5422, 5647, 4132, and 2074 proteins were aligned to the Swiss-Prot, Pfam, GO, KEGG, and COG databases, respectively. In addition, 2579 proteins were predicted with a transmembrane region and 1098 with a signal peptide. Complete results are shown in Supplementary Table S1-S3. According to the Swiss-Prot annotation innexin unc-9 (13 in *R. viridisi* and 11 in *S. longicornis*), FMRFamide receptor (6 and 6), innexin unc-7 (5 and 8), uncharacterized protein K02A2.6 (13 and 24), FMRFamide-activated amiloride-sensitive sodium channels (5 and 7), histone-lysine N-methyltransferase SETMAR (6 and 12), Tigger transposable element-derived protein 6 (7 and 7), potassium voltage-gated channel protein Shaw (4 and 7), cathepsin L/L1 (3 and 8) were among the most abundant proteins with annotation. The UniProt KeyWords transferase (36.7% in *R. viridisi* and 42.1% in *S. longicornis*), virulence (23.8% and 19.7%), DNA-binding (16.5% and 12.4%), and ANK repeat (8.8% and 10.2%) were the most abundant annotated (Supplementary Table S7).

The prediction indicated that the annotated proteins are vinculated, at level four, mainly with phenotypes such as cytokinesis variant emb (3.6% in *R. viridisi* and 4.2% in *S. longicornis*), muscle arm development defective (3.2% and 2.6%), sexually dimorphic development variant (2.7% and 2.5%), carbon dioxide response variant (2.8% and 2.9%), cell proliferation increased (2.2% and 2.5%), developmental morphology variant (2.1% and 1.9%), negative chemotaxis variant (2% and 2.3%), and drug-induced gene expression variant (2.1% and 1.7%). At level three, organelle organization biogenesis variant (5.5% and 6.3%), embryonic cell organization biogenesis variant (3.5% and 4.1%), egg-laying variant (3.3% and 2.9%), aldicarb response variant (3% and 2.4%), organism pathogen response variant (3.3% and 3.6%), cell fate specification variant (3.4% and 3.1%), chemotaxis variant (2.9% and 2.8%) and cell stress response variant (1.8% and 2.%) were the most represented worm phenotypes (Supplementary Table S3).
Among the most represented GO terms appeared ATP binding (4.5% in *R. viridisi* and 4.7% in *S. longicornis*), metal ion binding (3.9% and 3.8%), protein binding (3.7% and 3.4%), DNA binding (2.8% and 2.7%), RNA binding (2.3% and 2.3%), nucleic acid binding (1.9% and 1.6%), and calcium ion binding (1.8% and 1.5%), protein kinase activity (1.2% and 1%), protein serine/threonine kinase activity (1% and 1%), and G protein-coupled receptor activity (1% and 0.6%) within the Molecular Function (Supplementary Figure S1); protein phosphorylation (0.6% and 0.6%), regulation of transcription, DNA-templated (0.8% and 0.6%), positive regulation of transcription by RNA polymerase II (0.6% and 0.7%), transmembrane transport (0.6% and 0.6%), and G protein-coupled receptor signaling pathway (0.6% and 0.4%) within the Biological Process (Supplementary Figure S2); and nucleus (7.2% and 7.1%), cytoplasm (6.1% and 6.9%), integral component of membrane (5.1% and 4.9%), cytosol (4.7% and 4.5%), plasma membrane (4.8% and 4.3%), and membrane (3.4% and 3%) within the Cellular Component (Supplementary Figure S3).
The KEGG annotations showed that membrane trafficking [BR:ko04131] (3.4% in *R. viridisi* and 3.1% *S. longicornis*), chromosome and associated proteins [BR:ko03036] (3.3% and 3.1%), transporters [BR:ko02000] (2.1% and 1.8%), exosome [BR:ko04147] (2% and 1.8%), protein kinases [BR:ko01001] (1.9% and 1.4%), messenger RNA biogenesis [BR:ko03019] (1.7% and 1.5%), ion channels [BR:ko04040] (1.7% and 1.4%) and peptidases and inhibitors [BR:ko01002] (1.1% and 1.1%) were among the main metabolic pathways in which the likely proteins are involved (Supplementary Figure S4).
The most representative Pfam domains were protein kinase domain (2% and 1.8%), protein tyrosine and serine/threonine kinase (2% and 1.8%), leucine rich repeat (1.5% and 1.2%), 7 transmembrane receptor (rhodopsin family) (1.4% and 1.1%), immunoglobulin domain (1.2% and 1.1%), tetratricopeptide repeat (1.2% and 1.1%), WD domain, G-beta repeat (1.1% and 1.1%), RNA recognition motif. (a.k.a. RRM, RBD, or RNP domain) (1.1% and 1.1%), and ankyrin repeat (0.7% and 1.2%) (Supplementary Table S8).
Among the most representative COG terms were serine threonine protein kinase (5.6% and 4.6%), major facilitator Superfamily (4.2% and 3.1%), phosphatidylinositol kinase activity (3.6% and 2.5%), and calcium-binding protein (3.5% and 3.1%) (Supplementary Table S9).

Lineage-specific GPCR

To identify lineage-specific GPCR, the predicted GPCRs of *R. viridisi* and *S. longicornis* (Queries) were aligned against the NCBI non-redundant protein (nr) databases (Subject) limited by the option “Organism”, which included the sister classes, Cestoda (taxid:6199), Trematoda (taxid:6178); the basal class, Rhabditophora (taxid:147100) and the taxa

Lophotrochozoa (taxid:1206795) (exclude: Platyhelminthes), Spiralia (taxid:2697495) (exclude: Lophotrochozoa), Protostomia (taxid:33317) (exclude: Spiralia), Bilateria (taxid:33213) (exclude: Protostomia) and Vertebrata (taxid:7742). 16 (eighth of *R. viridisi* and 8 of *S. longicornis*) dataset of e-values were obtained. Those predicted GPCRs with e-value >1e^{-05}, detected through heatmaps using the ggtree package in R-studio (R version 4.0.4) ggtree library (Yu et al., 2017), were considered to be specific for Monogenea. According to Kerfeld and Scott (2011) “sequences with a recent shared ancestry will have a high degree of similarity; their alignments will have many identical residues, few substitutions and gaps, and tiny e-values; conversely, sequences with an ancient common ancestor will be deeply divergent, with few shared sequence identities, many gaps, and larger e-values'', therefore, considering that sequences with hits can be interpreted as sequences sharing evolutionary history, the e-values were correlated between different datasets through Spearmen analysis. For instance, all e-values of the alignment of GPCRs of *R. viridisi* (Query) and Trematoda (Subject) were correlated with the e-values obtained by aligning GPCRs of *R. viridisi* (Query) against GPCRs of Cestoda (Subject) (Supplementary figure). Similar evolutionary patterns among taxa are expected to result in correlation values close to 1, whereas in those taxas where the selective pressure is different, for example due to adaptive issues, correlation values far away 1 may be expected.
We propose that the correlation values can be used to explore evolutionary patterns. Proteins with low mutational rate, usually essential proteins, show lower e-values even between distant taxa, whereas those with a higher mutation rate, usually proteins related to adaptive processes, tend to show higher e-values.

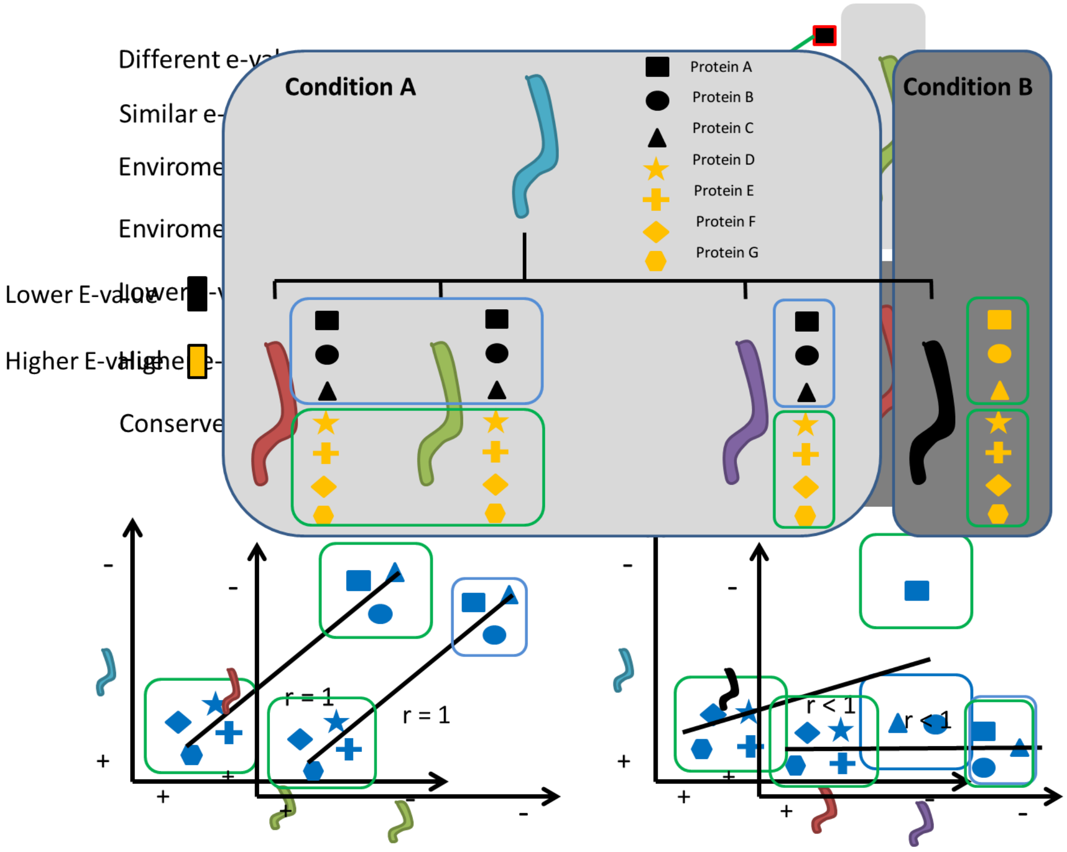

Code Availability

Assembly:
Trimmomatic (v0.35) R1_001.fastq.gz R2_001.fastq.gz Paired_R1.fastq.gz Unpaired_R1.fastq.gz PairedR2.fastq.gz R2.fastq.gz ILLUMINACLIP:all_adapters.fa:2:30:10 SLIDINGWINDOW:4:15 LEADING:5 TRAILING:5 AVGQUAL:20 MINLEN:40
Trinity (v2.8.6) --seqType fq --max_memory 100G --left Paired_R1.fastq --right PairedR2.fastq --SS_lib_type RF --CPU 32 --normalize_by_read_set
Filtered of contaminant sequences of the assembly
makeblastdb (Blast v2.7.1) -in assembly_trinity.fasta -out assembly_trinity.fasta -parse_seqids -dbtype nucl
blastx (Blast v2.7.1) -query assembly_trinity.fasta -db uniprot_Bacter.fasta -outfmt "6 qacc sacc qcovs qcovhsp pident evalue bitscore length" -evalue 0.00001 -out transcriptome_Bacter
fastagrep.pl (https://github.com/rec3141/rec-genome-tools) -X -f code_transcriptome_Bacter assembly_trinity.fasta > code_transcriptome_Bacter.fasta
makeblastdb (Blast v2.7.1) -in uniprot_Gyro_Enip_Hmicro_Sman_Smed_Bacter.fasta -out uniprot_Gyro_Enip_Hmicro_Sman_Smed_Bacter -parse_seqids -dbtype prot
blastx (Blast v2.7.1) -db uniprot_Gyro_Enip_Hmicro_Sman_Smed_Bacter -query code_transcriptome_Bacter.fasta -outfmt "6 qacc sacc qcovs qcovhsp pident evalue bitscore length" -evalue 0.00001 -max_target_seqs 1 -out best_hit_bac
makeblastdb -in assembly_trinity_without_bacter.fasta -out assembly_trinity_without_bacter.fasta -parse_seqids -dbtype nucl
blastx -query assembly_trinity_without_bacter.fasta -db fish_host.pep -outfmt "6 qacc sacc qcovs qcovhsp pident evalue bitscore length" -evalue 0.0000000000000000001 -out _transcriptome_Host
fastagrep.pl -X -f code_transcriptome_Host assembly_trinity.fasta > code_transcriptome_Host.fasta
makeblastdb -in Gyro_Enip_Hmicro_Sman_Smed_Fish.fasta -out Gyro_Enip_Hmicro_Sman_Smed_Fish.fasta -parse_seqids -dbtype prot
blastx -db Gyro_Enip_Hmicro_Sman_Smed_Fish.fasta -query code_transcriptome_Host.fasta -outfmt "6 qacc sacc qcovs qcovhsp pident evalue bitscore length" -evalue 0.00001 -max_target_seqs 1 -out best_hit_fish
makeblastdb -in assembly_trinity_without_bacter_fish.fasta -out assembly_trinity_without_bacter_fish.fasta -parse_seqids -dbtype nucl
blastx -query assembly_trinity_without_bacter_fish.fasta -db uniprot_virus_fungi.fa -outfmt "6 qacc sacc qcovs qcovhsp pident evalue bitscore length" -evalue 0.00001 -out _transcriptome_virus_fungi
fastagrep.pl -X -f code_transcriptome_virus_fungi assembly_trinity.fasta > code_transcriptome_virus_fungi.fasta
makeblastdb -in uniprot_Gyro_Enip_Hmicro_Sman_Smed_virus_fungi.fasta -out uniprot_Gyro_Enip_Hmicro_Sman_Smed_virus_fungi.fasta -parse_seqids -dbtype prot
blastx -db uniprot_Gyro_Enip_Hmicro_Sman_Smed_virus_fungi.fasta -query code_transcriptome_virus_fungi.fasta -outfmt "6 qacc sacc qcovs qcovhsp pident evalue bitscore length" -evalue 0.00001 -max_target_seqs 1 -out best_hit_virus_fungi
Prediction of ORFs
TransDecoder.LongOrfs (v5.5) -t assembly_trinity_without_bacter_fish_virus_fungi.fasta
blastp (Blast v2.7.1) -query longest_orfs.pep -db uniprot_sprot.pep -max_target_seqs 1 -outfmt 6 > blastp.outfmt6
hmmscan (v3.2.1) --cpu 10 --domtblout TrinotatePFAM.out Pfam-A.hmm longest_orfs.pep > pfam.log
TransDecoder.Predict (v5.5) -t assembly_trinity_without_bacter_fish_virus_fungi.fasta --retain_pfam_hits TrinotatePFAM.out --retain_blastp_hits blastp.outfmt6
Cluster of ORFs

cd-hit (v4.6) -i assembly_trinity_without_bacter_fish_transdecoder.pep -o unigene.pep -c 1.00

Filtered of contaminant sequences of the Rabdo ORF

makeblastdb -in vibrio_spp_platyhelminthes.fasta -out vibrio_spp_platyhelminthes.fasta -parse_seqids -dbtype prot

blastp -db vibrio_spp_platyhelminthes.fasta -query rhabdo_unigene.pep -outfmt "6 qacc sacc qcovs qcovhsp pident evalue bitscore length" -evalue 0.00001 -max_target_seqs 1 -out best_hit_vibrio

makeblastdb -in robalo_lates_tilapia_platyhelminthes.fasta -out robalo_lates_tilapia_platyhelminthes.fasta -parse_seqids -dbtype prot

blastp -db robalo_lates_tilapia_platyhelminthes.fasta -query rhabdo_unigene.pep -outfmt "6 qacc sacc qcovs qcovhsp pident evalue bitscore length" -evalue 0.00001 -max_target_seqs 1 -out best_hit_vibrio

Annotation

blastx -query assembly_trinity_without_bacter_fish_virus_fungi.fasta -db uniprot_sprot.pep -max_target_seqs 1 -evalue 0.00001 -outfmt 6 > blastx.outfmt6

RnammerTranscriptome.pl (v3.1.1) --transcriptome assembly_trinity_without_bacter_fish_virus_fungi.fasta --path_to_rnammer ~/bin/RNAMMER/rnammer

signalp (v4.1) -f short -n signalp.out assembly_trinity_without_bacter_fish_virus_fungi.fasta.transdecoder.pep

tmhmm (v2.0) --short <assembly_trinity_without_bacter_fish_virus_fungi.fasta.transdecoder.pep> tmhmm.out

get_Trinity_gene_to_trans_map.pl (v3.1.1) assembly_trinity_without_bacter_fish_virus_fungi.fasta > assembly_trinity_without_bacter_fish_virus_fungi.fasta.gene_trans_map

Trinotate (v3.1.1) Trinotate.sqlite init --gene_trans_map assembly_trinity_without_bacter_fish_virus_fungi.fasta.gene_trans_map --transcript_fasta assembly_trinity_without_bacter_fish_virus_fungi.fasta. --transdecoder_pep unigene.pep

Trinotate Trinotate.sqlite LOAD_swissprot_blastp ../blastp.outfmt6

Trinotate Trinotate.sqlite LOAD_swissprot_blastx ../blastx.outfmt6

Trinotate Trinotate.sqlite LOAD_pfam TrinotatePFAM.out

Trinotate Trinotate.sqlite LOAD_tmhmm tmhmm.out

Trinotate Trinotate.sqlite LOAD_signalp signalp.out

Trinotate Trinotate.sqlite LOAD_rnammer tmp.superscaff.rnammer.gff

Trinotate Trinotate.sqlite report > Trinotate.xls

awk '{print $5"\t"$2"\t"$3"\t"$4"\t"$1"\t"$6"\t"$7"\t"$8"\t"$9"\t"$10"\t"$11"\t"$12"\t"$13"\t"$14"\t"$15}'| sed 's/_____/ /g' Trinotate.xls > Trinotate_unigenes.xls

trinotate_report_summary.pl (v3.1.1) Trinotate_unigenes.xls report_trinotate.out

Mapped

bowtie2-build (v2.3.4) assembly_trinity.fasta assembly_mapping

bowtie2 (v2.3.4) --no-unal -x assembly_mapping -1 _Paired_R1.fastq.gz -2 _Paired_R2.fastq.gz -S assembly_mapping.sam.gz

samtools (v1.7) view -bSo assembly_mapping.bam.gz assembly_mapping.sam.gz

samtools sort assembly_mapping.bam.gz -o assembly_mapping_sorted.bam

samtools index assembly_mapping_sorted.bam

samtools depth Rhabdo_contig.sorted.bam | awk '{sum+=$3} END { print "Average = ",sum/NR}' > OutFile.coverage

Evaluation of the transcriptome

python run_BUSCO.py (v.3.0.2) -i unigene.pep -o unigene -l metazoa_odb9 -m prot
