# Supplementary material for: De novo transcriptome assembly and identification of G-Protein-Coupled-Receptors (GPCRs) in two species of monogenean parasites of fish
Source: Parasite. 2022 Nov 9;29:51. doi: 10.1051/parasite/2022052 (PMC9645230; doi:10.1051/parasite/2022052)
Supplement: Supplementary file 2 — – Supplementary Table S1. Annotation of putative proteins of Rhabdosynochus viridisi. [file parasite-29-51-s2.pdf]

Table 1. Assembly and ORF statistics generated for *Rhabdosynochus viridisi* and *Scutogyrus longicornis*.

	S. longicornis							***R. viridisi***						
	Unfiltered	First filtering		Second filtering		Third filtering		Unfiltered	First filtering		Second filtering		Third filtering	
	Assembly	Assembly	ORF	Assembly	ORF	Assembly	ORF	Assembly	Assembly	ORF	Assembly	ORF	Assembly	ORF
# sequences ≥0 bp	51817	49873	22197	48775	21241	47814	19291	294928	269985	55141	267005	47497	264494	41059
# sequences ≥1000 bp	20258	19127	6663	14119	6464	13169	5997	63638	54716	8818	52818	7067	51163	5670
# sequences ≥3000 bp	10291	9594	455	5361	445	4852	408	33686	28665	678	23965	560	20032	458
# sequences	82366	78594	29315	68255	28150	65835	25696	392252	353366	64637	343788	55124	335689	47187
# genes	48086	46372	13179	45995	12821	45194	12020	313109	284085	30291	280648	26854	277651	23857
Total length (bp)	111966103	105481027	23584962	70992660	22763481	66069113	20893392	484764415	422751986	40917888	369417136	34017756	329849780	28346136
GC (%)	42.56	42.49	46.38	42.49	46.44	42.46	4649	45.72	45.75	51.2	45.34	51.27	44.92	51.35
Average length of sequences	1359.37	1342.1	804.54	1040.11	808	1003.56	813	1235.85	1196.36	633.04	1074	617	982	600
N50	2906	2874	1020	2197	1029	2114	1038	3567	3496	750	2636	711	2132	666

Table 2. Information on contaminating sequences in the transcriptomes of *Rhabdosynochus viridisi* and *Scutogyrus longicornis*.

Filter		Contaminant taxa	Number of contaminant sequences	% GC	Average sequence	Number of bases
First filtering (assembly)	*S. longicornis* contigs (first filtering)	Bacteria	292	47.77	1362.50	397850
		Tilapia	3285	43.16	1776.21	5834843
		Viruses and fungi	195	48.27	1319.91	258703
	R. viridisi contigs (first filtering)	Bacteria	11284	45.25	2085.79	23536059
		Snooks	27133	45.50	1364.26	37016466
		Viruses and fungi	469	49.28	3112.80	1459904
Second filtering (ORF)	*S. longicornis* ORF (second filtering)	Bacteria	55	45.80	682.80	37554
		Tilapia	1110	44.66	706.24	783927
	R. viridisi ORF	*Vibrio* spp.	2071	45.99	621.04	1286169
		Snooks and tilapia	7442	51.93	754.36	5613963
Third filtering (ORF)	*S. longicornis* ORF (third filtering)	Non-Protostomia	2454	45.81	762.06	1870089
	R. viridisi ORF (third filtering)	Non-Protostomia	7937	50.9	714.58	5671620

Table 3. Numbers of top-hits of *Rhabdosynochus viridisi* and *Scutogyrus longicornis* ORFs matching to sequences of other species. Information was obtained from a similarity search analysis using TRAPID and the EggNOG database. Top hits obtained with sequences from other Platyhelminthes are shown.

***R. viridisi* unfiltered ORF**		***R. viridisi* filtered ORF (first filtering: bacteria+fish)**		***R. viridisi* filtered ORF (second filtering: bacteria+fish)**		***S. longicornis unfiltered ORF***		***S. longicornis* filtered ORF (first filtering: bacteria+fish)**		***S. longicornis* filtered ORF (second filtering: bacteria+fish)**		***G. salaris***		***E. nipponicum***		***P. xenopodis***		***S. mediterranea***	
Hits	23274	Hits	16448	Hits	11108	Hits	15417	Hits	14031	hits	12273	Hits	8699	Hits	16499	Hits	11353	Hits	21189
Sm	11297 (48.54%)	*Sm*	9547 (58.04%)	*Sm*	7321 (65.91%)	*Sm*	8880 (57.6%)	Sm	8247 (58.78%)	*Sm*	7854 (63.99%)	*Sm*	4865 (55.93%)	*Sm*	10583 (64.14%)	*Sm*	8327 (73.35%)	*Sm*	5756 (27.17%)
On	699 (3%)	*Bf*	455 (2.77%)	*Bf*	310 (2.79%)	*Bf*	466 (3.02%)	Bf	414 (2.95%)	*Bf*	330 (2.69%)	*Bf*	224 (2.58%)	*Bf*	417 (2.53%)	*Bf*	253 (2.23%)	*Bf*	1505 (7.1%)
Bf	608 (2.61%)	*Sp*	259 (1.57%)	*Sp*	174 (1.57%)	*Sp*	402 (2.61%)	Sp	377 (2.69%)	*Sp*	266 (2.17%)	*Sp*	123 (1.41%)	*Sp*	275 (1.67%)	*Sp*	199 (1.75%)	*Sp*	819 (3.87%)
Ga	431 (1.85%)	*On*	249 (1.51%)	*Dp*	135 (1.22%)	*Ap*	269 (1.74%)	Ap	214 (1.53%)	*Ap*	186 (1.52%)	*Dr*	119 (1.37%)	*Dp*	203 (1.23%)	*Nv*	107 (0.94%)	*Hm*	625 (2.95%)
Sp	396 (1.7%)	*Ga*	183 (1.11%)	*Is*	134 (1.21%)	*Tc*	214 (1.39%)	Tc	209 (1.49%)	*Tc*	185 (1.51%)	*Dp*	117 (1.34%)	*Nv*	192 (1.16%)	*Is*	104 (0.92%)	*Tc*	616 (2.91%)
Vs	353 (1.52%)	*Dr*	183 (1.11%)	*Tc*	117 (1.05%)	*Hm*	212 (1.38%)	Hm	201 (1.43%)	*Dp*	157 (1.28%)	*Tc*	116 (1.33%)	*Dr*	172 (1.04%)	*Dp*	99 (0.87%)	*Dp*	528 (2.49%)

Abbreviations: *Ap*, *Acyrthosiphon pisum*; *Bf*, *Branchiostoma floridae*; *Dp*, *Daphnia pulex*; *Dr*, *Danio rerio*; *Ga*, *Gasterosteus aculeatus*; *Hm*, *Hydra magnipapillata*; *Is*, *Ixodes scapularis*; *Nv*, *Nematostella vectensis*; On, *Oreochromis niloticus*; *Sm*, *Schistosoma mansoni*; Sp, *Strongylocentrotus purpuratus*; Tc, *Tribolium castaneum*; Vs, *Vibrio sinaloensis*.

Table 4. Identification and classification of GPCR obtained from different platyhelminths.

GPCR family	Monogenea						Trematoda			Cestoda		Rhabditophora	
	Monopisthocotylea				Polyopisthocotylea		Digenea			Eucestoda		Seriata	
	R. viridisi	*S. longicornis*	*G. bullatarudis*	*G. salaris*	*P. xenopodis*	*E. nipponicum*	*S. japonicum*	*S. mansoni*	*F. hepatica*	*T. asiatica*	*E. multilocularis*	*B. semperi*	*S. mediterranea*
Adhesion/secretin	2/1	1/1	5	4		7	6	7		4	4	13	27
Frizzled	6	3	9	9	9	2	7	6	5	5	6	7	11
Glutamate	1	3	3	2	3		4	3	3	6	2	2	10
Rhodopsin	99	94	82	83	73	14	100	102	97	64	58	45	336
**Total**	**110**	**102**	**99**	**98**	**85**	**23**	**117**	**118**	**105**	**79**	**70**	**67**	**384**
