# Supplementary material for: De novo transcriptome assembly and identification of G-Protein-Coupled-Receptors (GPCRs) in two species of monogenean parasites of fish
Source: Parasite. 2022 Nov 9;29:51. doi: 10.1051/parasite/2022052 (PMC9645230; doi:10.1051/parasite/2022052)
Supplement: Supplementary file 3 — – Supplementary Table S2. Annotation of putative proteins of Scutogyrus longicornis. [file parasite-29-51-s3.pdf]

Table 2. Information on contaminating sequences in the transcriptomes of *Rhabdosynochus viridisi* and *Scutogyrus longicornis*.

Filter		Contaminant taxa	Number of contaminant sequences	% GC	Average sequence	Number of bases
First filtering (assembly)	*S. longicornis* contigs (first filtering)	Bacteria	292	47.77	1362.50	397850
		Tilapia	3285	43.16	1776.21	5834843
		Viruses and fungi	195	48.27	1319.91	258703
	R. viridisi contigs (first filtering)	Bacteria	11284	45.25	2085.79	23536059
		Snooks	27133	45.50	1364.26	37016466
		Viruses and fungi	469	49.28	3112.80	1459904
Second filtering (ORF)	*S. longicornis* ORF (second filtering)	Bacteria	55	45.80	682.80	37554
		Tilapia	1110	44.66	706.24	783927
	R. viridisi ORF	*Vibrio* spp.	2071	45.99	621.04	1286169
		Snooks and tilapia	7442	51.93	754.36	5613963
Third filtering (ORF)	*S. longicornis* ORF (third filtering)	Non-Protostomia	2454	45.81	762.06	1870089
	R. viridisi ORF (third filtering)	Non-Protostomia	7937	50.9	714.58	5671620
