# Supplementary material for: De novo transcriptome assembly and identification of G-Protein-Coupled-Receptors (GPCRs) in two species of monogenean parasites of fish
Source: Parasite. 2022 Nov 9;29:51. doi: 10.1051/parasite/2022052 (PMC9645230; doi:10.1051/parasite/2022052)
Supplement: Supplementary file 4 — – Supplementary Table S3. UniProt keyword annotation of putative proteins of Rhabdosynochus viridisi and Scutogyrus longicornis. – Supplementary Table S4. Information on each step of the classification of GPCRs of Rhabdosynochus viridisi and Scutogyrus longicornis. – Supplementary Table S5. Information on GPCR identification in each platyhelminth species. – Supplementary Table S6. Information on the e-values of the alignment of GPCRs. – Supplementary Table S7. UniProt annotation of putative proteins of Rhabdosynochus viridisi and Scutogyrus longicornis. – Supplementary Table S8. Domain annotation of putative proteins of Rhabdosynochus viridisi and Scutogyrus longicornis. – Supplementary Table S9. COG annotation of putative proteins of Rhabdosynochus viridisi and Scutogyrus longicornis. [file parasite-29-51-s4.zip › parasite220026-4-olm/parasite220026-4-olm.pdf]

Table 3. Numbers of top-hits of *Rhabdosynochus viridisi* and *Scutogyrus longicornis* ORFs matching to sequences of other species. Information was obtained from a similarity search analysis using TRAPID and the EggNOG database. Top hits obtained with sequences from other Platyhelminthes are shown.

***R. viridisi* unfiltered ORF**		***R. viridisi* filtered ORF (first filtering: bacteria+fish)**		***R. viridisi* filtered ORF (second filtering: bacteria+fish)**		***S. longicornis unfiltered ORF***		***S. longicornis* filtered ORF (first filtering: bacteria+fish)**		***S. longicornis* filtered ORF (second filtering: bacteria+fish)**		***G. salaris***		***E. nipponicum***		***P. xenopodis***		***S. mediterranea***	
Hits	23274	Hits	16448	Hits	11108	Hits	15417	Hits	14031	hits	12273	Hits	8699	Hits	16499	Hits	11353	Hits	21189
Sm	11297 (48.54%)	*Sm*	9547 (58.04%)	*Sm*	7321 (65.91%)	*Sm*	8880 (57.6%)	Sm	8247 (58.78%)	*Sm*	7854 (63.99%)	*Sm*	4865 (55.93%)	*Sm*	10583 (64.14%)	*Sm*	8327 (73.35%)	*Sm*	5756 (27.17%)
On	699 (3%)	*Bf*	455 (2.77%)	*Bf*	310 (2.79%)	*Bf*	466 (3.02%)	Bf	414 (2.95%)	*Bf*	330 (2.69%)	*Bf*	224 (2.58%)	*Bf*	417 (2.53%)	*Bf*	253 (2.23%)	*Bf*	1505 (7.1%)
Bf	608 (2.61%)	*Sp*	259 (1.57%)	*Sp*	174 (1.57%)	*Sp*	402 (2.61%)	Sp	377 (2.69%)	*Sp*	266 (2.17%)	*Sp*	123 (1.41%)	*Sp*	275 (1.67%)	*Sp*	199 (1.75%)	*Sp*	819 (3.87%)
Ga	431 (1.85%)	*On*	249 (1.51%)	*Dp*	135 (1.22%)	*Ap*	269 (1.74%)	Ap	214 (1.53%)	*Ap*	186 (1.52%)	*Dr*	119 (1.37%)	*Dp*	203 (1.23%)	*Nv*	107 (0.94%)	*Hm*	625 (2.95%)
Sp	396 (1.7%)	*Ga*	183 (1.11%)	*Is*	134 (1.21%)	*Tc*	214 (1.39%)	Tc	209 (1.49%)	*Tc*	185 (1.51%)	*Dp*	117 (1.34%)	*Nv*	192 (1.16%)	*Is*	104 (0.92%)	*Tc*	616 (2.91%)
Vs	353 (1.52%)	*Dr*	183 (1.11%)	*Tc*	117 (1.05%)	*Hm*	212 (1.38%)	Hm	201 (1.43%)	*Dp*	157 (1.28%)	*Tc*	116 (1.33%)	*Dr*	172 (1.04%)	*Dp*	99 (0.87%)	*Dp*	528 (2.49%)

Abbreviations: *Ap*, *Acyrthosiphon pisum*; *Bf*, *Branchiostoma floridae*; *Dp*, *Daphnia pulex*; *Dr*, *Danio rerio*; *Ga*, *Gasterosteus aculeatus*; *Hm*, *Hydra magnipapillata*; *Is*, *Ixodes scapularis*; *Nv*, *Nematostella vectensis*; On, *Oreochromis niloticus*; *Sm*, *Schistosoma mansoni*; Sp, *Strongylocentrotus purpuratus*; Tc, *Tribolium castaneum*; Vs, *Vibrio sinaloensis*.
