# Supplementary material for: De novo transcriptome assembly and identification of G-Protein-Coupled-Receptors (GPCRs) in two species of monogenean parasites of fish
Source: Parasite. 2022 Nov 9;29:51. doi: 10.1051/parasite/2022052 (PMC9645230; doi:10.1051/parasite/2022052)
Supplement: Supplementary file 4 — – Supplementary Table S3. UniProt keyword annotation of putative proteins of Rhabdosynochus viridisi and Scutogyrus longicornis. – Supplementary Table S4. Information on each step of the classification of GPCRs of Rhabdosynochus viridisi and Scutogyrus longicornis. – Supplementary Table S5. Information on GPCR identification in each platyhelminth species. – Supplementary Table S6. Information on the e-values of the alignment of GPCRs. – Supplementary Table S7. UniProt annotation of putative proteins of Rhabdosynochus viridisi and Scutogyrus longicornis. – Supplementary Table S8. Domain annotation of putative proteins of Rhabdosynochus viridisi and Scutogyrus longicornis. – Supplementary Table S9. COG annotation of putative proteins of Rhabdosynochus viridisi and Scutogyrus longicornis. [file parasite-29-51-s4.zip › parasite220026-4-olm/parasite220026-5-olm.pdf]

Table 4. Identification and classification of GPCR obtained from different platyhelminths.

GPCR family	Monogenea						Trematoda			Cestoda		Rhabditophora	
	Monopisthocotylea				Polyopisthocotylea		Digenea			Eucestoda		Seriata	
	R. viridisi	*S. longicornis*	*G. bullatarudis*	*G. salaris*	*P. xenopodis*	*E. nipponicum*	*S. japonicum*	*S. mansoni*	*F. hepatica*	*T. asiatica*	*E. multilocularis*	*B. semperi*	*S. mediterranea*
Adhesion/secretin	2/1	1/1	5	4		7	6	7		4	4	13	27
Frizzled	6	3	9	9	9	2	7	6	5	5	6	7	11
Glutamate	1	3	3	2	3		4	3	3	6	2	2	10
Rhodopsin	99	94	82	83	73	14	100	102	97	64	58	45	336
**Total**	**110**	**102**	**99**	**98**	**85**	**23**	**117**	**118**	**105**	**79**	**70**	**67**	**384**
